# Deciphering the differences of bacterial communities between high- and low-productive wheat fields using high-throughput sequencing

**DOI:** 10.3389/fmicb.2024.1391428

**Published:** 2024-09-04

**Authors:** Hongjin Niu, Min Yuan, Xiaobo Chen, Jingwei Zhao, Yushuang Cui, Yao Song, Sihao Zhou, Alin Song, Yali Huang

**Affiliations:** ^1^School of Environmental Science and Engineering, Hebei University of Science and Technology, Shijiazhuang, China; ^2^College of Life Sciences, North China University of Science and Technology, Tangshan, China; ^3^College of Food Science and Biology, Hebei University of Science and Technology, Shijiazhuang, China; ^4^Institute of Agricultural Resources and Regional Planning, Chinese Academy of Agricultural Sciences, Beijing, China

**Keywords:** wheat, Illumina HiSeq sequencing, bacterial community, network analysis, mantel test

## Abstract

Microbial communities have been demonstrated to be essential for healthy and productive soil ecosystems. However, an understanding of the relationship between soil microbial community and soil productivity levels is remarkably limited. In this study, bulk soil (BS), rhizosphere soil (RS), and root (R) samples from the historical high-productive (H) and low-productive (L) soil types of wheat in Hebei province of China were collected and analyzed by high-throughput sequencing. The study highlighted the richness, diversity, and structure of bacterial communities, along with the correlation networks among different bacterial genera. Significant differences in the bacterial community structure between samples of different soil types were observed. Compared with the low-productive soil type, the bacterial communities of samples from the high-productive soil type possessed high species richness, low species diversity, complex and stable networks, and a higher relative abundance of beneficial microbes, such as *Pseudoxanthomonas*, unclassified Vicinamibacteraceae, *Lysobacter*, *Massilia*, *Pseudomonas*, and *Bacillus*. Further analysis indicated that the differences were mainly driven by soil organic matter (SOM), available nitrogen (AN), and electrical conductivity (EC). Overall, the soil bacterial community is an important factor affecting soil health and crop production, which provides a theoretical basis for the targeted regulation of microbes in low-productivity soil types.

## 1 Introduction

Wheat is the second most widely grown crop across the world, with 200 million hectares under cultivation, and is a staple food for approximately 35 to 40% of the population globally, providing 20% of calories and protein in the human diet. Wheat plays an important role in the food supply of many countries across the world (Husenov et al., 2021). In China, wheat, as the main food crop, is planted in an area of 23.57 million hectares, accounting for approximately 25% of the country’s total grain production. Increasing wheat yield is of great significance in stabilizing food security for China and even for the world (Li et al., 2023). Despite the widespread cultivation of wheat in China, its production is hindered mostly by soil fertility exhaustion, agricultural deterioration, and unfavorable cropping circumstances. Low soil fertility is considered to be the main constraint to wheat production and yield in China (Liu C. L. et al., 2015; Duan et al., 2016; Li J. et al., 2019).

Hebei Province is one of the largest agricultural production regions in China, accounting for 9.48% of China’s winter wheat planting area and 10.72% of China’s total grain production (Ren D. et al., 2018; Zhang et al., 2023). However, 60% of the grain fields in Hebei province are low-productive soil types, which seriously restricts the increase in the total wheat yield (Yan et al., 2016). The fertility of the soil has a direct impact on plant growth, either by physically influencing root growth and exposure to the soil solution or indirectly by regulating mineralization, nutrient retention in the soil, and the association between soil and plant water (Li et al., 2016; Chi et al., 2021). Additionally, fertile and productive soil sustains a diverse and dynamic community of biota, which contributes to nutrient cycling and retention and the preservation of soil structure (Pellegrino et al., 2020).

The fertility of soil can be compromised by the interplay of physical, chemical, and biological factors, ultimately leading to a detrimental effect on the development of crops (Xiao et al., 2022). While physical and chemical indicators of soil are commonly used to assess soil quality by farmers and researchers, biological indicators are often considered underrepresented (Buckland et al., 2018). However, the soil bacteria, as a biological indicator, play a crucial role in the creation and reinforcement of soil aggregates, leading to improved water infiltration, root penetration, and nutrient mobility, thus enhancing the soil structure (Guo et al., 2018; Belimov et al., 2022; Rabbi et al., 2022; Lu et al., 2023). An appropriately structured soil accelerates root growth and absorption of nutrients in plants, hence enhancing soil fertility. On the other hand, several studies have demonstrated that there is a mutual influence between soil chemical properties, microbial activity, and community structure (Rousk et al., 2010; Fanin and Bertrand, 2016; Veldkamp et al., 2020). Fan et al. found that the relative abundances of Actinobacteria, Chloroflexi, and Rokubacteria significantly decreased with increasing levels of desertification, whereas the opposite trend was detected for Proteobacteria (especially Alphaproteobacteria and Gammaproteobacteria) and Bateroidetes (Fan et al., 2020). As described previously, soil microbiota plays a crucial role in sustainable agriculture and crop production; combining chemical and microbiological indicators may be a good approach to characterize soil fertility gradients (Philippot et al., 2023).

Bacterial communities in soil, rhizosphere, and root are essential for the health of the soil and plant growth. However, the bacterial communities in different soil zones may be different in response to the same environmental factors. The abundance and diversity of bacteria were significantly developed by chemical fertilizer inputs in the rhizosphere compared with those in the bulk soil (Xiao et al., 2024). The addition of nitrogen significantly reduced bacterial diversity in the phyllosphere, rhizosphere soil, and bulk soil samples but not in the root endophytes and altered the community composition of bacteria and fungi in all four compartments. Cultivars could affect the community composition of root-associated bacteria and fungi. Soil saline also had an effect on the microbial community of bulk and rhizosphere soils than root endophytes (Sun et al., 2021). Although many studies showed that environmental factors, such as fertilizer input, saline, drought, and plant cultivars, have different effects on the microbial communities of different soil types, there are few reports on the specific relationship between soil fertility levels and the microbial community from the bulk soil, rhizosphere soil, and root samples. Identifying the reduced beneficial microflora and the enriched harmful microflora in the low-productive soil type can serve as a focal point for altering soil microflora and establishing a scientific basis for fostering healthy soil conditions, thereby enhancing wheat production. To clarify the relationship between soil fertility types and soil microbial characteristics, based on the crop yield statistics for 10 consecutive years, we collected samples from the historical high- and low-productive soil types of wheat in Hebei Province, performed bacterial 16S rRNA amplicon sequencing, and analyzed the bacterial communities in the current study.

## 2 Materials and methods

### 2.1 Description of the experimental area

The study area is located within Hebei province in China (36°05′N–42°40′N, 113°27′E–119°50′E). The average annual temperature in the province is 10–20°C, and the average annual precipitation is 484.5 mm. The planting area of wheat in Hebei province is 2.23 million hectares, with the proportion of high-productive fields being 27.6% and low-productive fields being 35.8%. The high- and low-productive fields were determined by the crop yield statistics for 10 consecutive years.

### 2.2 Sample collection

**Figure 1:**
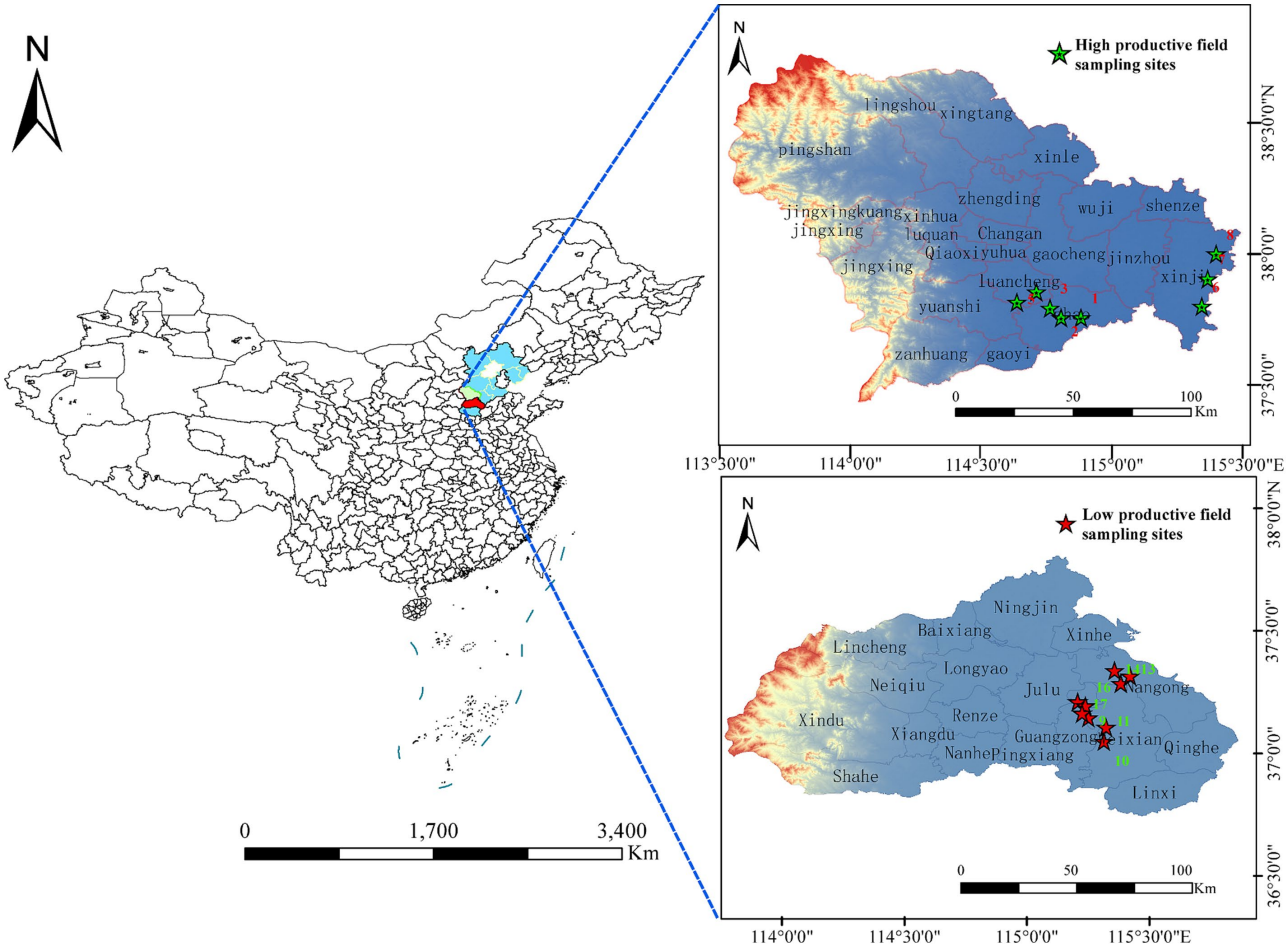
Map of the study sites located in Hebei province, which is in the northeast of China. Eight farmlands with high productivity and nine farmlands with low productivity were included in the study.

In May 2022, we randomly selected 17 wheat farmlands from 6 counties in Hebei province, which is located in the northeast of China. Eight farmlands with high productivity and nine farmlands with low productivity were selected in this study. High-productive fields were located in Zhao county, Luancheng county, and Xinjin county, respectively. Low-yield fields were located in Guangzong county, Wei county, and Nangong county, respectively (Hebei Provincial Bureau of Quality and Technical Supervision, 2021). The average annual yield of wheat in high- and low-productive fields in this study is 9,000–10,500 and 6,750–8,250 kg·ha^−1^, respectively. Randomly selected 2–3 wheat farmlands with a straight-line distance of more than 10 km are considered as sample points in each county and designated them as biological replicates. In each sampling plot, five wheat plants were collected using an “S” pattern and combined into one sample (Figure 1). The whole wheat root was completely uprooted with a shovel, which was re-sterilized between sample plots and transported to the laboratory in a sterile sealing bag on ice. The bulk soil samples (BS) were collected using a shaking method. The rhizosphere soil samples(RS)were collected using a sterilized brush to accumulate the soil adhered to the surface of fine roots, which had a thickness of approximately 1–2 mm. For root samples (R), the roots were washed three times using sterile water and then sonicated in a sterilized tube for 3 min at 60 Hz (sonication for 60 s, break for 30 s), to remove the microbes from the rhizoplane. After removing litter, stones, and soil earthworms, all bulk soil samples were sieved through a 2-mm mesh and divided into two parts: one part was stored at −80°C for microorganism analysis and the other part was air-dried for the determination of soil chemical properties. The rhizosphere soil samples and root samples were stored at −80°C for microorganism analysis.

### 2.3 Sample analyses

#### 2.3.1 Soil chemical analyses

The analyses of bulk soil chemical properties were conducted as follows: The pH was measured by a pH meter (PB-10, Beijing, China), and electrical conductivity was measured by a conductivity meter (DDS-307A, Shanghai Leici, China), with a soil to water ratio of 1: 2.5 (w/v). Soil available phosphorus (AP), available potassium (AK), available nitrogen (AN), and soil organic matter (SOM) were determined as previously described (Yan et al., 2021). Principal component analysis (PCA) of soil chemical properties was analyzed by R (version 4.4.2) (Qin et al., 2022).

#### 2.3.2 DNA extraction, PCR amplification, and Illumina HiSeq sequencing

The total DNA of each sample was extracted using the PowerSoil DNA Isolation Kit. The bacterial 16S rRNA V3–V4 region of each sample was amplified using primers 338F (5′-ACT CCT ACG GGA GGC AGC A-3′) and 806R (5′-GGA CTA CHV GGG TWT CTA AT-3′). The PCR products were purified with a DNA gel extraction kit (Axygen, Shanghai, China) and verified by 1.8% agarose gel electrophoresis. Finally, an Illumina HiSeq 2,500 platform (Illumina, Inc., San Diego, CA, United States) was used to perform high-throughput sequencing at Biomarker Technologies Corporation (Beijing, China). Raw image data files obtained in the current study were transformed into the original sequence reads using base calling analysis. The sequence information and corresponding sequencing quality information were stored in FASTQ (fq) file format. All sequences were deposited in the NCBI Sequence Read Archive with the BioProject ID PRJNA 1081352.

#### 2.3.3 Statistical and bioinformatics analyses

Trimmomatic (version 0.33) was used to filter the raw reads obtained from sequencing, with parameters of SLIDINGWINDOW:50:20 and MINLEN:215. Then, cutadapt (version 1.9.1) was used to obtain clean reads by removing primer sequences. Then, raw FASTQ files were further processed using QIIME software (version 1.8.0) for demultiplexing, quality filtering, and data analysis. The high-quality sequences were obtained by filtering the raw tags using FLASH (Magoc and Salzberg, 2011) and were further clustered using DADA2. The tags were calculated using QIIME for bacterial α-diversity analysis (Callahan et al., 2016; Bolyen et al., 2019). Non-metric multidimensional scaling (NMDS) of bacterial β-diversity was performed at the ASV level based on the Bray_Curtis algorithm (Looft et al., 2012). Kruskal–Wallis test in the linear discriminant analysis (LDA) effect size (LEfSe) method was performed to detect the potential indicators. The taxa with significant differences between low- and high-productive soil samples were determined by LDA ≥ 3.5 and *p* < 0.05 (Segata et al., 2011). SPSS 17.0 was used to test differences in soil chemical properties and bacterial α-diversity indices between low- and high-productive soil samples using the independent samples t-test. The co-occurrence network of bacterial communities was constructed according to the relative abundance files at the genus level. The genera were filtered based on the abundance size and the correlation, which was calculated using the Spearman correlation coefficient. In this study, the genera with abundance of >0.1%, correlation of >0.1, and *p*-value of <0.05 were selected to construct the network. R software was used to transform the relative abundance table at the genus level into a correlation coefficient matrix. Then, Gephi 0.10.1 software was used to visualize the network based on the correlation coefficient matrix. Ultimately, the role of nodes was determined and classified according to the connectivity among modules (Pi) and connectivity within modules (Zi). The nodes with Zi < 2.5 and Pi ≥0.62 are categorized as connectors (key taxa) (Deng et al., 2012). The Mantel test was used to analyze the interrelationship between soil chemical properties and bacterial communities.

## 3 Results

### 3.1 Soil chemical properties

**Figure 2:**
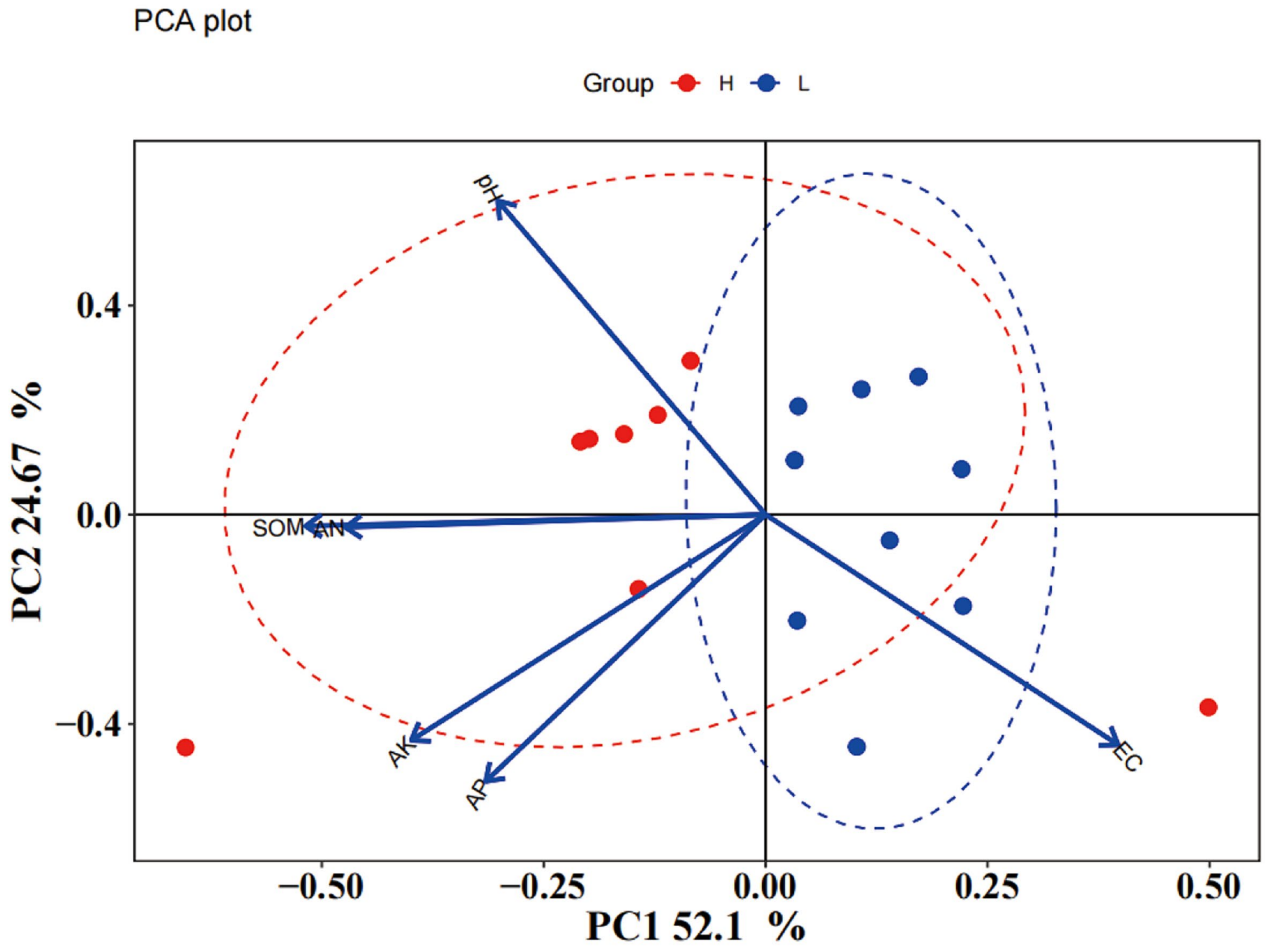
Principal component analysis (PCA) of soil properties.

Soil chemical properties usually serve as an important indicator of soil fertility type. In this study, the chemical properties of the high- and low-productive soil type samples were determined. The contents of SOM, AN, and AP in the low-productive soil type samples were significantly lower than those in the high-productive soil type samples, but the soil EC was higher than that in the high-productive soil type samples (*p* < 0.05). However, there were no significant differences in pH and AK contents among the different types of soils (Table 1). Principal component analysis (PCA) showed that two-dimensional PCA for soil chemical properties could explain 76.77% of the total variance of soil fertility types of all samples, and the chemical properties showed obvious changes along the first axis of PCA, with the high-productive soil type samples on the left and the low-productive soil type samples on the right, indicating a good differentiation between the two soil type samples in terms of soil chemical properties (Figure 2).

**Table 1:** Soil chemical properties of the high- and low-productive soil type samples.

Groups	SOM/ (g·kg^−1^)	AN/ (mg·kg^−1^)	AP/ (mg·kg^−1^)	AK/ (mg·kg^−1^)	EC/ (μS·cm^−1^)	pH
H	26.56 ± 1.15^*^	153.43 ± 5.97^*^	21.73 ± 1.55^*^	169.51 ± 13.76	267.00 ± 7.76	7.82 ± 0.11
L	16.71 ± 1.39	83.15 ± 14.24	12.63 ± 1.67	173.35 ± 14.89	553.50 ± 48.83^*^	7.72 ± 0.10

H, high-productive soil type samples; L, low-productive soil type samples; SOM, Soil Organic Matter; AN, Available Nitrogen; AP, Available Phosphorus; and AK, Available Potassium. The results were given as mean ± SD (standard deviation). Statistical significances were determined by the Independent-samples T Test (^*^p < 0.05).

### 3.2 Richness and diversity of the bacterial community

**Figure 3:**
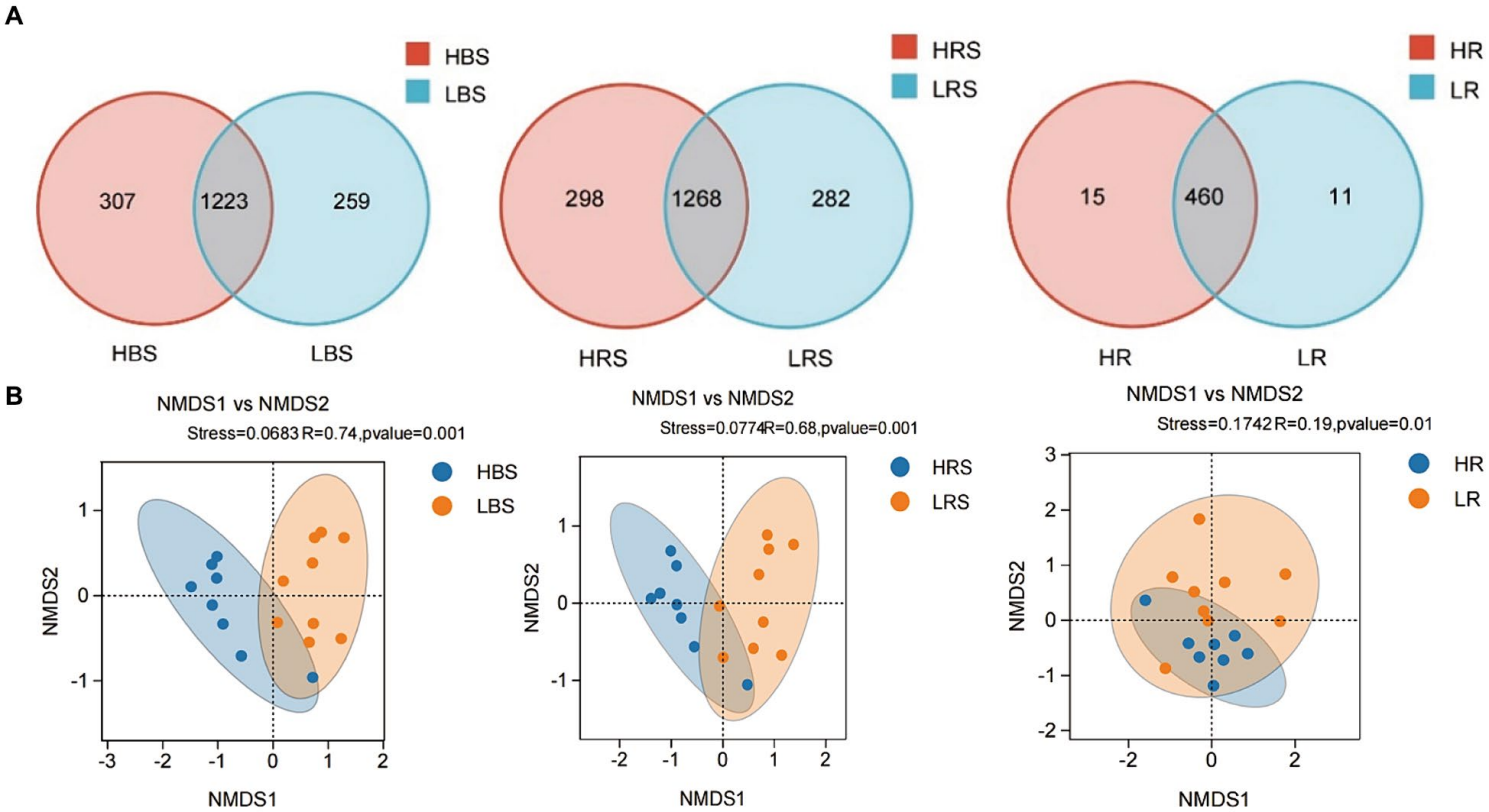
**(A)** Venn diagram of the composition of bacterial communities in the BS, RS, and R samples. Different colors represented different treatments. The numbers in overlapping and non-overlapping sections referred to the quantity of common ASVs and unique ASVs of samples from different types of soil. **(B)** Non-Metric Multi-Dimensional Scaling (NMDS) analysis of the high-productive and low-productive soil type groups in the bulk soil, rhizosphere soil, and root samples, respectively. The scatter plot of 8 samples from the high-productive group and 9 samples from the low-productive soil type group represented the bacterial ASV community composition. The distance between points represented the degree of difference based on unweighted UniFrac similarities in each group.

A total of 1,547,961 effective sequences were obtained in the bacterial community analysis of 51 samples. The coverage value of each sample was higher than 99.99%, implying sufficient sequencing depths for assessing bacterial biodiversity in all samples. Bacterial communities were analyzed by comparing the ASVs (ASV quantities), diversity indices (Shannon and Simpson), and richness indices (ACE and Chao1) between low- and high-productive soil types within bulk soil, rhizosphere soil, and root samples, respectively. Although no significant differences were observed between low- and high-productive soil type samples, a larger number of ASVs were obtained in the high-productive soil type samples than in the low-productive soil type samples within bulk soil, rhizosphere soils, and root soil groups (Table 2). Meanwhile, a Venn diagram was used to show the differences in the bacterial community based on unique and shared ASVs across the two groups with each subgroup. The numbers of shared ASVs by the two different groups within BS, RS, and R subgroups were 1,223, 1,268, and 460, respectively. The numbers of unique ASVs of the low-productive soil type samples in the BS, RS, and R subgroups (259, 282, and 11) were lower than those of the high-productive soil type samples (307, 298, and 15), indicating that the species richness decreased in the low-productive soil type (Figure 3A).

**Table 2:** Richness and diversity of the high- and low-productive soil types samples.

	Samples	Sequence numbers	ASVs	ACE	Chao1	Simpson	Shannon	Coverage/%
BS	HBS	39,726 ± 1,284	644 ± 30	645.27 ± 30.48	646.68 ± 31.95	0.9967	8.76 ± 0.08	99.99
	LBS	38,020 ± 2,786	635 ± 71	636.46 ± 71.34	637.76 ± 72.44	0.9967	8.78 ± 0.18	99.99
RS	HRS	43,182 ± 2,261	677 ± 55	678.90 ± 55.38	680.21 ± 55.93	0.9963	8.76 ± 0.19	99.99
	LRS	37,721 ± 2699**	645 ± 66	646.31 ± 66.43	649.86 ± 65.66	0.9968	8.79 ± 0.18	99.99
R	HR	13,260 ± 1775	263 ± 31	263.39 ± 31.13	263.42 ± 31.07	0.9934	7.65 ± 0.20	99.99
	LR	10,773 ± 1153**	239 ± 48	239.07 ± 48.51	238.98 ± 48.54	0.9923	7.48 ± 0.40	99.99

The results were presented as mean ± SD (standard deviation). Statistical significances were determined by the independent samples *t*-test. The differences between low- and high-productive soil types within bulk soil, rhizosphere soil, and root samples are indicated by * for *p* < 0.05 and ** for *p* < 0.01.

### 3.3 Bacterial community β-diversity

To visualize the differences in community structure between high- and low-productive soil type samples, NMDS and analysis of similarities (ANOSIM) were conducted at the ASV level. The results showed that the eight repetitions from the high-productive group and the nine repetitions from the low-productive group were clustered together, respectively, indicating that the bacterial community structure in this study had good representativeness (Figure 3B). In the bulk soil and rhizosphere soil samples, a clear separation was observed between high- and low-productive soil type samples within each sample type (bulk soil: R = 0.74, *p* = 0.001; rhizosphere soil: R = 0.68, *p* = 0.001). However, in the root samples, the degree of separation between bacterial communities decreased (root: R = 0.19, *p* = 0.01). These results indicated that the soil microbial community structure of bacteria has a strong relation with soil fertility.

### 3.4 Bacterial community composition

**Figure 4:**
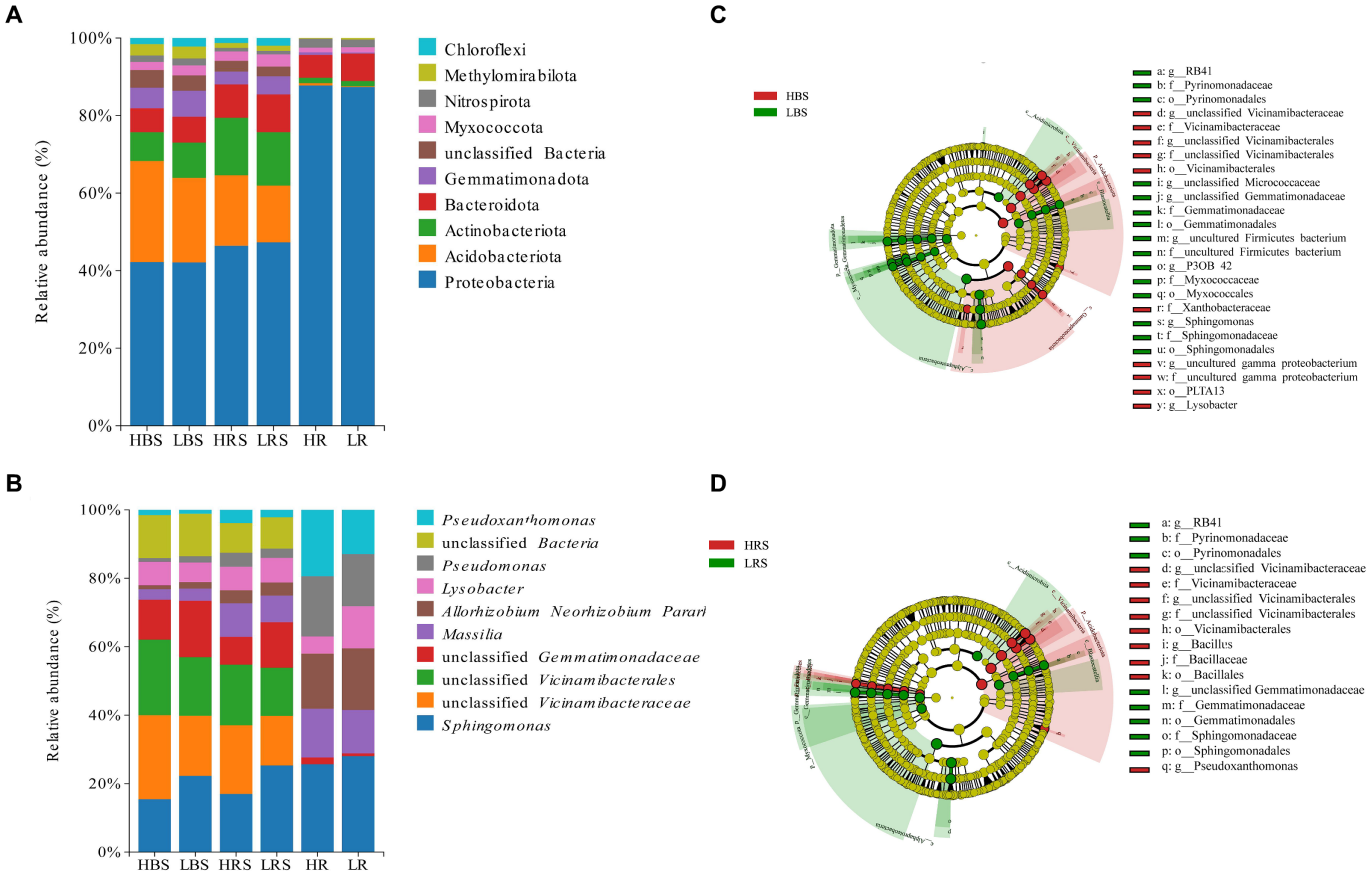
Relative abundances on **(A)** phylum and **(B)** genus levels of bacteria of different soil and root samples. Cladogram based on LEfSe analysis (LDA > 3.5) of the **(C)** bulk soil samples bacterial community and **(D)** rhizosphere soil samples, showing that bacteria that significantly differ between groups, color-coded in red and green. The classification levels from phylum to genus are organized concentrically, with the innermost circle representing phylum and the outermost representing genus. The yellow circle represents ASVs with no significant difference.

The microbial community structure in the bulk soil, rhizosphere soil, and root samples was analyzed at the bacterial phylum level in both high- and low-productive soil type samples. In the bulk soil samples, the abundance and order of the top 10 dominant phyla were similar, with the highest abundance of Proteobacteria, Acidobacteriota, Actinobacteriota, Bacteroidota, and Gemmatimonadota, accounting for more than 85% of the overall relative abundance. Among the high abundance of phyla, Gemmatimonadota was significantly higher in the low-productive soil type samples than in the high-productive soil type samples. Moreover, among the low-abundance phyla, the relative abundance of Dadabacteria, Desulfobacterota, and Campylobacterota was significantly higher than that of the high-productive soil type samples (Supplementary Table S1). The dominant phyla in the rhizosphere soil samples were the same as in the bulk soil samples, and there was no significant difference in the abundance in the dominant phyla of soil bacteria between high- and low-productive soil type samples, but Firmicutes were highly significantly different (*p* < 0.001) with abundances of 0.75% in the high-productive soil type samples and 0.10% in the low-productive soil type samples (Supplementary Table S3). In the root samples, Proteobacteria were highly enriched with an abundance of 85.57 to 86.04%, followed by Bacteroidota, and comparative analyses of all bacterial phyla of the two productive soil type samples revealed that none of the bacterial groups showed significant differences (Supplementary Table S5; Figure 4A). From the above analyses, it can be observed that the differences between bacterial community decreased sequentially from bulk soil and rhizosphere soil to root samples at different levels of productivity.

At the genus level, the dominant genera in the bulk soil samples were similar at both high- and low-productive soil type samples, with *Sphingomonas*, unclassified Vicinamibacteraceae, *Allorhizobium*, *Massilia*, *Lysobacter*, *Pseudoxanthomonas*, *Pseudomonas,* and unclassified Gemmatimonadaceae. Among them, the relative abundance of *Sphingomonas* and unclassified Gemmatimonadaceae in the bulk soil samples of the low-productive soil type samples was significantly increased, while the relative abundance of unclassified Vicinamibacteraceae, *Pseudoxanthomonas*, and *Lysobacter* significantly decreased (*p* < 0.01) (Supplementary Table S2). Analysis of bacterial community composition at the genus level and abundance of the rhizosphere soil samples between high- and low-productive soil type samples revealed that the significant transformation of bacteria in the low-productive soil type samples compared to the high-productive soil type samples was the same as in the bulk soil samples, with unclassified Gemmatimonadaceae and *Sphingomonas* increasing by 38.86 and 27.68%, respectively. *Pseudoxanthomonas*, unclassified Vicinamibacteraceae, and *Lysobacter* were decreased by 53.74, 37.83, and 12.81%, respectively (Supplementary Table S4). Analysis of microbial community composition within the root samples revealed that the abundance of bacterial community within the root samples varied considerably from the soil samples, with *Lysobacter* increasing and *Massilia* and *Pseudomonas* significantly decreasing in the root samples of the low-productive soil type samples (Supplementary Table S6; Figure 4B).

### 3.5 Indicator bacteria for two productive soil type samples

Indicator bacteria are usually treated as specialized communities that represent microbial communities with statistically significant differences. In the bulk soil samples, there are more potential biomarkers in the low-productive soil type samples than those in the high-productive soil type samples; the phylum Proteobacteria together with its three main orders (Rhizobiales, Xanthomonadaceae, and PLTA13) and the order Vicinamibacterales and its genus unclassified Vicinamibacterales (phylum Acidobacteriota) were enriched in the HBS, while the family Pyrinomonadaceae together with its order Pyrinomonadales, order Myxococcales (phylum Myxococcota), order Gemmatimonadales (phylum Gemmatimonadota), and order *Sphingomonas* (Proteobacteria) was dominant in the LBS (Figure 4C). In addition, in the rhizosphere soil samples, the relative abundance of the family Pyrinomonadaceae (phylum Acidobacteriota), order Gemmatimonadales (phylum Gemmatimonadota), and order Sphingomonadales (Proteobacteria) were also dominant in the LRS. However, in the HRS, the order Bacillales was specifically enriched together with its family Bacillaceae and the genus *Bacillus* (phylum Firmicutes) (Figure 4D).

### 3.6 Co-occurrence network structure of bacterial communities

**Figure 5:**
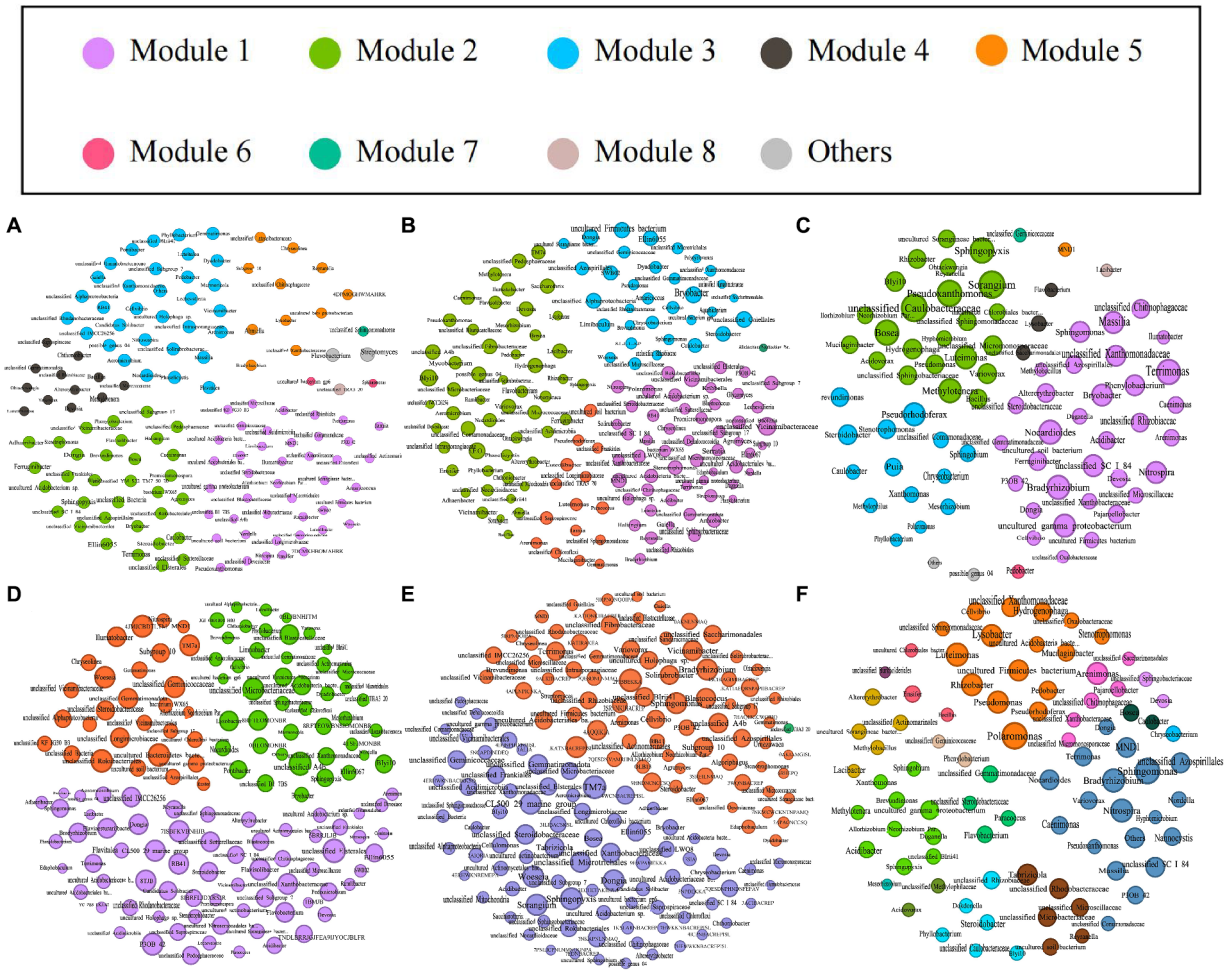
The networks of co-occurring bacterial in the bulk soils, rhizosphere soils, and root samples in both high- and low-productive soil type samples of six groups, **(A)** HBS, **(B)** HRS, **(C)** HR, **(D)** LBS, **(E)** LRS, and **(F)** HR, based on correlation analysis. The co-occurring networks are colored by module. Within each panel, the node size corresponds proportionally to its degree of connectivity.

We explored the bacterial co-occurrence patterns using network analysis. Six networks were comprised of bulk soil, rhizosphere soil, and root samples of the high- and low-productive soil type samples, respectively (Figure 5). The quantities of total nodes and total links in the low-productive soil type samples were higher than those in the high-productive soil type samples, except for the HR at total links. Additionally, the bacterial networks of the low-productive soil type samples had a lower average clustering coefficient than those of the high-productive soil type samples, indicating that the bacterial network of the low-productive soil type samples was simpler. The co-occurrence analyses for the bulk soil and rhizosphere soil samples showed an augment in graph density for the low-productive soil type samples, indicating that bacteria of the bulk soil and rhizosphere soil samples in low-productive soil type samples are closely connected. This may be due to lower module numbers in the low-productive soil type samples, resulting in lower average path lengths and greater susceptibility to external environmental factors. Moreover, the rhizosphere soil samples of the low-productive soil type samples have a higher number of negative links, indicating a more competitive correlation among bacteria in the LRS network. However, for the root samples of the low-productive soil type samples, the graph density was decreased, suggesting that the LR networks had prominent ‘small-world’ modularity and hierarchy of their topological properties. As a consequence, the inter-bacterial network of the bulk soil and rhizosphere soil samples in the low-productive soil type samples is simpler and more unstable. Additionally, the LR exhibited small-world characteristics, rendering a more efficient whole system in the root samples from the low-productive soil type samples (Table 3).

**Table 3:** Topological properties of co-occurring bacterial networks and their corresponding random networks.

Network metrics	BS		RS		R	
	HBS	LBS	HRS	LRS	HR	LR
Number of nodes	137	141	157	163	81	86
Number of edges	817	912	1,037	1,282	255	224
Number of positive correlations (%)	53.00	53.07	53.52	49.06	52.94	62.50
Number of negative correlations (%)	47.00	46.93	46.48	50.94	47.06	37.50
Average path length (APL)	2.812	2.616	2.651	2.571	3.576	3.539
Graph density	0.088	0.092	0.085	0.097	0.079	0.061
Network diameter	6	5	6	6	9	8
Average clustering coefficient (avg CC)	0.451	0.419	0.422	0.417	0.47	0.478
Number of modules	10	3	5	3	10	16
Modularity (M)	6.256	6.510	6.114	−273.055	6.330	1.839

Based on Spearman’s correlations, we further selected the genus with a correlation of >0.6 and a *p*-value of <0.05 and analyzed the microbial networks of bacteria in different groups. The results showed that a few nodes were designated as a connector in the respective networks, and the connectors were defined as key taxa (Zi ≤ 2.5, Pi > 0.62). In the bulk soil samples, the high-productive soil type samples had two connectors, both belonging to the Proteobacteria, while the low-productive soil type samples had four connectors. In addition to Proteobacteria, Chloroflexi (as one of the main carbon-fixing phyla) and Bacteroidota were determined to be connectors in the low-productive soil type samples. This phototrophic mode is a stress-resistant strategy under nutrient-deficiency conditions, indicating that the low-productive soil type samples need to include other phyla to enhance bacterial interactions. In contrast, in the rhizosphere soil samples, there were four connectors in both high- and low-productive soil type samples. Compared with the low-productive soil type samples where all connectors are Proteobacteria, the high-productive soil type samples also include Acidobacteria and Bacteroidetes in their connectors, with Bacteroidetes being the primary degraders of complex carbohydrate biomass. In the root samples, the connectors of both high- and low-productive soil type samples are Proteobacteria. In summary, there are significant differences in key species at different productivity levels, which are mainly manifested in the bulk soil and rhizosphere soil samples, and these two ecological niches have different nutritional availability and needs (Table 4).

**Table 4:** Nodes identified as connectors of bacterial networks (HBS, LBS, HRS, LRS, HR, and LR).

Genus	Role	Abundance (%)	Degree	Phyla	Zi value	Pi value
**HBS**						
Unclassified Xanthobacteraceae	Connector	0.0139	9	Proteobacteria	0.4975	0.6400
Steroidobacter	Connector	0.0086	19	Proteobacteria	0.4954	0.6400
**LBS**						
Unclassified Chloroflexi	Connector	0.0093	13	Chloroflexi	0.0000	0.6400
Sphingomonas	Connector	0.0693	17	Proteobacteria	0.4902	0.6667
Terrimonas	Connector	0.0079	17	Bacteroidota	1.1180	0.6563
Unclassified SC I 84	Connector	0.0060	16	Proteobacteria	−1.1180	0.6250
**HRS**						
Unclassified Comamonadaceae	Connector	0.0076	20	Proteobacteria	−0.8492	0.6250
Unclassified Vicinamibacteraceae	Connector	0.0619	31	Acidobacteriota	0.8235	0.6250
Pedobacter	Connector	0.0096	7	Bacteroidota	−0.4611	0.6667
Unclassified Xanthobacteraceae	Connector	0.0088	12	Proteobacteria	−0.8018	0.7222
**LRS**						
Cellvibrio	Connector	0.0056	26	Proteobacteria	−1.0394	0.6250
BIyi10	Connector	0.0062	15	Proteobacteria	0.4103	0.6667
Altererythrobacter	Connector	0.0212	6	Proteobacteria	0.0000	0.6250
**HR**						
Altererythrobacter	Connector	0.0268	7	Proteobacteria	0.0000	0.6667
Bradyrhizobium	Connector	0.0138	12	Proteobacteria	1.1674	0.6667
Pseudorhodoferax	Connector	0.0092	11	Proteobacteria	0.3529	0.6627
Pseudomonas	Connector	0.0593	7	Proteobacteria	0.0000	0.6250
**LR**						
Cellvibrio	Connector	0.0167	6	Proteobacteria	−0.4590	0.6250
Altererythrobacter	Connector	0.0316	5	Proteobacteria	1.2585	0.6250
Bradyrhizobium	Connector	0.0130	11	Proteobacteria	1.1893	0.6400
Sphingopyxis	Connector	0.0100	5	Proteobacteria	0.0000	0.6667
Acidibacter	Connector	0.0164	5	Proteobacteria	0.0000	0.6667

The topological importance of each node is determined by two attributes: the within-module connectivity Zi, which gauges how strongly a node is connected to others within its module, and the among-module connectivity Pi, which measures the extent of a node’s connections to nodes in different modules.

### 3.7 Relationships between bacterial communities and soil chemical properties

**Figure 6:**
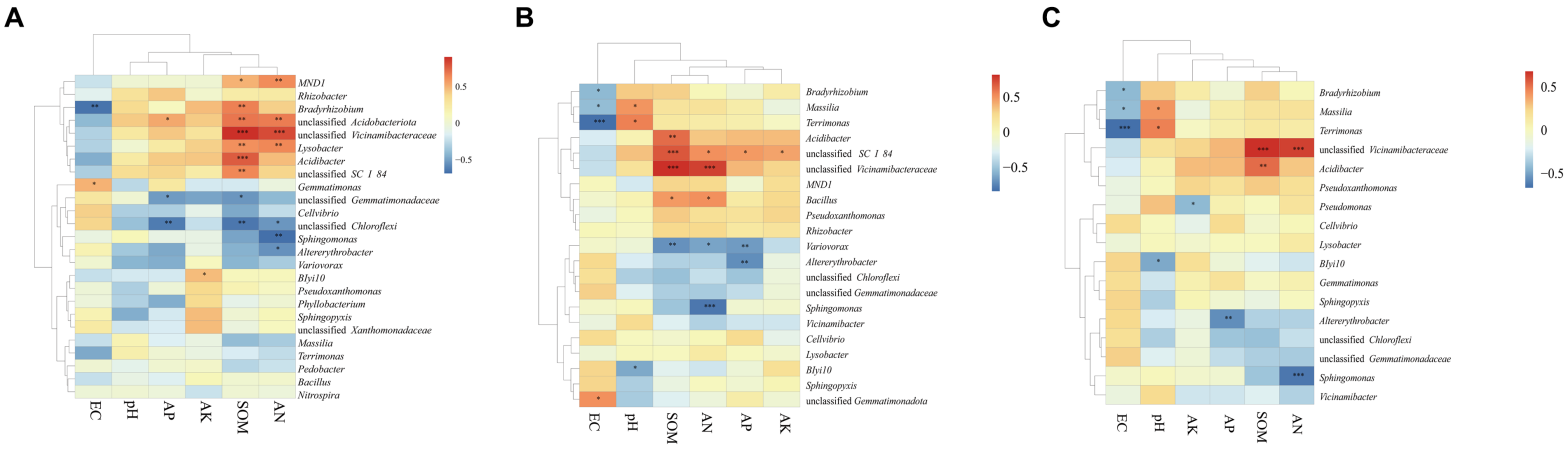
Correlation-based heatmap method reveals the relation between environmental factors and bacterial genera in the **(A)** BS, **(B)** RS, and **(C)** R. The different colors and intensities were adjusted based on associations among traits.

The Mantel test was performed to assess the relationship between bacterial communities and environmental factors, including six chemical properties: pH, EC, SOM, AN, AP, and AK. The changes in the bacterial community structure between high- and low-productive soil type samples in the bulk soil and rhizosphere soil samples were associated with SOM (bulk soil: R = 0.767, *p* = 0.001; rhizosphere soil: R = 0.683, *p* = 0.001), AN (bulk soil: R = 0.699, *p* = 0.001; rhizosphere soil: R = 0.608, *p* = 0.001), and soil EC (bulk soil: R = 0.325, *p* = 0.019; rhizosphere soil: R = 0.361, *p* = 0.010) (Table 5). In addition, the heatmap was used to further analyze the correlation between environmental factors and bacterial genera. In the bulk soil and rhizosphere soil samples, unclassified Gemmatimonadaceae and *Sphingomonas* showed significant negative correlations with AN, respectively. Unclassified Vicinamibacteraceae and *Lysobacter* exhibited a significant positive correlation with SOM and AN. In the root samples, a significant negative correlation was found between *Massilia* and EC. From the above analyses, it can be observed that soil fertility indices such as SOM, AN, and EC were the main factors affecting the soil bacterial community. The interaction between soil fertility index and bacteria would determine the soil productivity (Figure 6).

**Table 5:** Mantel analysis of environmental factors and bacterial genera.

Environment	BS		RS		R	
	R	P	R	P	R	P
pH	0.102	0.195	0.168	0.139	0.016	0.435
EC	0.325	0.019	0.361	0.010	0.221	0.097
SOM	0.767	0.001	0.683	0.001	0.190	0.106
AN	0.699	0.001	0.608	0.001	0.129	0.167
AP	−0.051	0.586	−0.032	0.457	−0.069	0.570
AK	0.091	0.184	0.006	0.366	−0.021	0.446

## 4 Discussion

### 4.1 Correlations between microbial communities and soil properties

Soil microbes are vital components of agricultural ecosystems, and their abundance and diversity are correlated with soil fertility. Increasing the application of organic fertilizers significantly enhances both soil organic matter content and microbial diversity (Karami et al., 2012; Ai et al., 2015; Tkacz et al., 2015). Below-ground interactions by the maize–peanut intercropping increased the number of beneficial soil bacteria and diversity of bacterial communities (Simpson and Shannon indices), which were conducive to improving the supply capacity of soil nutrients (N and P) and the stability of the soil microbial ecosystems (Li et al., 2007). Continuous cropping, mono-nutrient depletion, and declining soil fertility led to a decrease in soil microbial abundance (Chao1 index) and diversity (Shannon index), which promoted the growth of soil harmful microorganisms (Zhao et al., 2016). In this study, compared with the high-productive soil type samples, the bacterial richness indices (ACE and Chao1) in the bulk soil, rhizosphere soil, and root samples in the low-productive soil type samples were lower than those in the high-productive soil type samples, indicating that the lack of nutrients in the low-productive soil type samples may affect the growth and abundance of certain microorganisms.

However, in the current study, the bacterial diversity indices (Shannon and Simpson) in the low-productive soil type samples were higher than those in the high-productive soil type samples. Similar results were also obtained from previous research studies. For example, the diversity of bacterial communities decreased due to the increase in the amount of soil nitrogen; the Shannon diversity index of inter-root soil bacterial communities in plants was higher under low nitrogen conditions than that at high nitrogen levels, probably due to the suppression of beneficial interactions between the microbiome and non-leguminous plants under high fertilizer conditions (Kavamura et al., 2018). In addition, Sun et al. (2015) showed that soil nutrient content was low in the no-fertilizer treatment for 30 consecutive years, and bacterial abundance (16S rRNA gene copy number) was correspondingly low but phylogenetic diversity (PD) was high, which was manifested by higher bacterial diversity and analyzed that soil pH was the strongest driver of bacterial diversity.

In summary, bacterial diversity is influenced by various factors. In certain situations, nutrient deficiency may lead to decreased bacterial richness. Conversely, under specific conditions where the proliferation of certain bacterial communities is suppressed, bacterial diversity might increase in the low-productive soil type samples.

### 4.2 Relation between soil chemical properties and microbial community structure

In recent years, several studies on the differences in microbial communities in various types of farmlands have been conducted. Bandara et al. compared the differences in root microbial communities between high-yield and low-yield sites in soybean farms in Pennsylvania. The high-yield sites had more root-colonizing bacteria that increase plant growth, and the soil conditions were not ideal for symbiotic nitrogen fixation in low-yield sites (Bandara et al., 2021). Our study further analyzed the bacterial community structures of the high- and low-productive soil type samples in Hebei province of China. The dominant bacterial phyla in different productive soil types were consistent, such as Proteobacteria, Acidobacteriota, Actinobacteria, Bacteroidota, and Gemmatimonadota. These bacterial phyla are common in soils of various habitats and play essential roles in nutrient cycling, such as carbon, nitrogen, and phosphorus cycling (Li Y. et al., 2019; Ibrahim et al., 2020; Xue et al., 2020; Yan et al., 2021). Among them, Proteobacteria exhibited the highest abundance and played a crucial role in nutrient cycling in environments rich in organic matter (Ren C. J. et al., 2018). Meanwhile, Actinobacteria and Bacteroidota played dominant roles in the decomposition of soil organic matter (Six et al., 2004). Gemmatimonadota play an important role in nutrient transformation. Gemmatimonadota can transform inorganic nutrient elements into organic forms through its metabolism, such as nitrate into amino acids (Ibrahim et al., 2020).

Although the dominant bacteria of the high- and low-productive soil type samples were similar, there were obvious differences in the abundance of some bacteria between the two soil fertility samples. Members of Acidobacteria, especially those in the family Pyrinomonadaceae and order Pyrinomonadales, order Myxococcales (phylum Myxococcota), order Gemmatimonadales (phylum Gemmatimonadota), and order Sphingomonadales (Proteobacteria) were significantly more abundant in the low-productive soil type samples compared to the high-productive soil type samples. Acidobacteria were considered oligotrophic microorganisms, which thrive in low-nutrient soil, leading to a high abundance in the low-productive soil type samples (Jackson et al., 2007). Certain groups within the phylum Gemmatimonadota can adapt to dry conditions. Given the low water retention capacity of the low-productive soil type samples due to the soil structure, this adaptation might explain the increased abundance of these groups (Yan et al., 2021). Additionally, there was a notable increase in the abundance of Sphingomonas (a class within Proteobacteria) in the low-productive soil type samples. This bacterium is among the top five root pathogens (Deng et al., 2022). Conversely, there was a significant decrease in the abundance of certain groups in the phylum Actinobacteria, such as those in the order Rhizobiales, the family Xanthomonadaceae, and the genera *Lysobacter* and *Pseudoxanthomonas*. The genus *Lysobacter* exhibited strong antagonistic activity against various plant pathogenic fungi, bacteria, and nematodes and became a new type of biocontrol bacteria with biocontrol potential (Hayward et al., 2010). Furthermore, in the rhizosphere soil samples of the low-productive soil type samples, the abundance of *Bacillus*, a genus within Firmicutes, was significantly lower than that in the high-productive soil type samples. Rhizobiales, which are capable of symbiotic nitrogen fixation in leguminous plants, leading to increased nitrogen absorption by plant roots, demonstrated higher abundance in the low-productive soil type samples (Masson-Boivin and Sachs, 2018). *Bacillus* are saprotrophic microorganisms, have preferable biocontrol and growth-promoting potential, and are a potential strain to control wheat root rot caused by *F. oxysporum* (Xiong et al., 2015). The abundance of these microorganisms serves as an indicator of the health of soil microbial communities. Kandasamy et al. (2019) found that introducing key microorganisms from high-productive soil type samples altered the original microbial characteristics in the low-productive soil type samples, thereby enhancing soil biological health and yield. These findings lay the foundation for constructing healthy microbial communities in the low-productive soil type samples and targeted the regulation of soil microbes.

### 4.3 Shifts of structure in co-occurrence network for different productive soil types

In ecological environments, microorganisms cannot survive in an isolated environment, and they form complex network systems through interactions (Liu J. J. et al., 2015; Liu et al., 2016). In these intricate ecosystems, microbial interactions are more crucial for ecological functionality than microbial abundance and diversity (Zhao et al., 2022a). In recent years, researchers predominantly focused on microbial diversity and community structure in ecosystems, neglecting interactions between microbial communities. However, the functionality of microbial communities in complex ecosystems was not analyzed comprehensively in these studies. Based on the differences between bacterial communities in wheat-planting soil and root samples from different soil types in Hebei province, the inter-species interactions were further analyzed in this study. Co-occurrence network analysis was used to explore the interactions within bacterial communities in the high- and low-productive soil type samples. The results indicated that compared with the high-productive soil type samples, the low-productive soil type samples exhibited lower average clustering coefficients, fewer modules, and longer average path lengths, indicating decreased complexity in bacterial coexistence networks. The complexity of the microbial symbiotic network in the ecosystem is influenced by many factors. Zheng et al. (2021) showed that the occurrence of *Ralstonia solanacearum* has reduced the complexity of its microbial network compared with that in healthy tobacco plants. Although this study did not statistically analyze the occurrence of wheat root rot disease in the low-productive soil type samples, the reduced complexity of the bacterial network to some extent may reflect the unhealthy condition of soil microbial communities in the low-productive soil type samples.

However, the reduced complexity of the microbial network does not necessarily imply weakened interactions among microbial communities. Zhao et al. (2022b) observed enhanced microbial interactions during the composting process despite the decreased complexity and diversity of microbial networks during high-temperature fermentation. Similarly, Yuan et al. (2021) found increased microbial interactions despite decreased microbial diversity with increasing temperatures. The current study also revealed higher average degrees in the co-occurrence networks of the bulk soil and rhizosphere soil samples in the low-productive soil type samples, indicating tighter connections among soil microbes. In addition, the LR exhibited small-world characteristics, which could allow the effects of a perturbation to distribute rapidly through the entire network, rendering a more efficient system, consistent with the findings by Yuan et al.

Key taxa within soil microbial network modules play a crucial role in maintaining the stability of functional microbial communities (Zhou et al., 2010). Due to their specific roles in different substance cycles in soil, these key taxa are vulnerable to disruption caused by changes in soil environments (Steele et al., 2011). Changing the hub species (that is, species that are associated with many other species) would impact the community structure, such as rebuilding microbial co-occurrence networks (Faust and Raes, 2012). Therefore, analyzing key taxa in different habitats is essential. Fan et al. (2019) found that long-term fertilization suppressed the growth of nitrogen-fixing microbial clusters (modules), leading to reduced soil nitrogen-fixing functionality and indicating that fertilizer regulation of soil ecological functions might occur through the growth of specific microbial modules. Similar conclusions were drawn in the research by Wagg et al. These studies indicate that higher microbial community diversity and more complex microbial network significantly increased soil ecosystem function related to carbon and nutrient cycling (Wagg et al., 2019). In this study, significant differences in key bacterial species were found between samples from the high- and low-productive soil types, especially in the bulk soil and rhizosphere soil samples, where the differences were more pronounced. Except for the Proteobacteria phylum (a key phylum in the high-productive soil type samples), other phyla microorganisms such as Chloroflexi and Bacteroidota were also served as connectors to increase the microbial interactions in the bulk soil samples from low-productive soil types. In contrast, in the rhizosphere soil and root samples, key species in the low-productive soil type samples belonged to Proteobacteria. The results indicated low capacities for specific enrichment of dominant keystone strains, and other genera microorganisms may be used to establish network interactions in the low-productive soil type samples due to a lack of key genera.

In summary, the enhanced understanding of soil bacterial communities will aid in regulating the balance of soil ecosystems and provide a theoretical reference for further targeted regulation mechanisms in the low-productive soil type samples. However, to further identify the direct impact of soil environmental variables on microbial communities, future research should consider trait-based approaches. For instance, dose–response relationships between soil variables and microbial growth should be quantified. Alternatively, modern multi-omics technology such as metagenomics, metatranscriptomics, and metabolomics can provide a better understanding of microbial communities and their functionality, revealing possibilities for manipulating microbial communities to enhance crop nutrient utilization efficiency and reduce fertilizer usage.

## Data availability statement

The names of the repository/repositories and accession number(s) can be found below: https://www.ncbi.nlm.nih.gov/sra/PRJNA1081352.

## Author contributions

HN: Writing – original draft, Conceptualization, Investigation, Software. MY: Writing – review & editing, Conceptualization. XC: Writing – review & editing, Investigation. JZ: Methodology, Writing – review & editing. YC: Investigation, Writing – review & editing. YS: Investigation, Writing – review & editing. SZ: Investigation, Writing – review & editing. AS: Writing – review & editing. YH: Writing – review & editing, Investigation, Methodology, Software.
