# Supplementary material for: Deciphering the differences of bacterial communities between high- and low-productive wheat fields using high-throughput sequencing
Source: Front Microbiol. 2024 Sep 4;15:1391428. doi: 10.3389/fmicb.2024.1391428 (PMC11408337; doi:10.3389/fmicb.2024.1391428)
Supplement: Supplementary file 1 [file Data_Sheet_1.pdf]

SUPPORTING INFORMATION

Deciphering the differences on bacterial communities between high- and low-productive wheat fields using high-throughput sequencing

Hongjin Niu[a,1], Min Yuan[b,1], Xiaobo Chen[c,1], Jingwei Zhao[a], Yushuang Cui[a], Yao Song[c], Sihao Zhou[a], Alin Song[d], Yali Huang[a,*]

[a] School of Environmental Science and Engineering, Hebei University of Science and Technology, Shi Jiazhuang, Hebei, 050018, PR China

[b] College of Life Sciences, North China University of Science and Technology, Tangshan, Hebei, 063210, PR China

[c] College of Food Science and Biology, Hebei University of Science and Technology, Shijiazhuang, Hebei, 050018, PR China

[d] Institute of Agricultural Resources and Regional Planning, Chinese Academy of Agricultural Sciences, Beijing 100081, PR China

* Corresponding author: School of Environmental Science and Engineering, Hebei University of Science and Technology, Shijiazhuang, Hebei 050018, PR China

E-mail address: huangyali2291@163.com (YH)

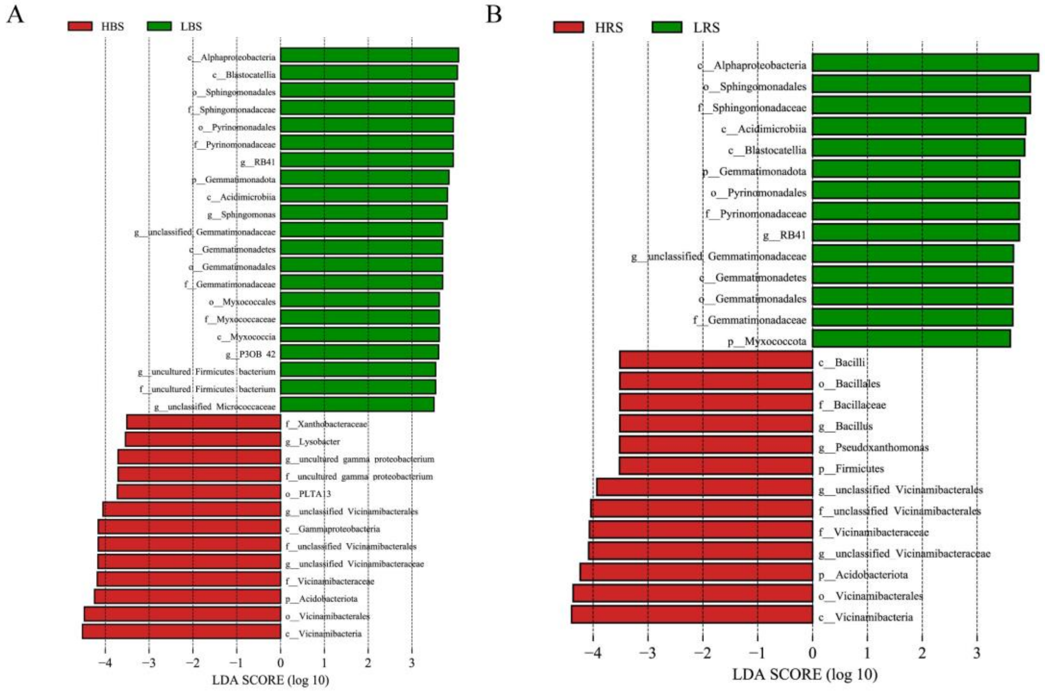

Figure S1

LEfSe analysis (LDA > 3.5) showed that bacterial community was significantly different between high and low-productive fields in the bulk soil samples (A) and rhizosphere soil samples (B), respectively. HBS: bulk soil samples (BS) from high-productive (H) fields; LBS: bulk soil samples (BS) from low-productive (L) fields; HRS: rhizosphere soil samples (RS) from high-productive (H) fields; LRS: rhizosphere soil samples (RS) from low-productive (L) fields.

Table S1. ANOVA analysis of variance for bulk soils on phylum level of bacteria.

phylum	LBS(Mean)	LBS(Sd)	HBS(Mean)	HBS(Sd)	MultiGroup(p)	MultiGroup(p-corrected)	LBS::HBS(p)
Proteobacteria	0.410445	0.015722	0.413596	0.014171	0.672053	0.739259	-
Acidobacteriota	0.212566	0.017512	0.254559	0.048326	0.027523	0.121101	-
Actinobacteriota	0.089337	0.028011	0.073437	0.042298	0.369996	0.478819	-
Bacteroidota	0.065214	0.009979	0.06007	0.010856	0.324659	0.476166	-
Gemmatimonadota	0.065012	0.009262	0.052067	0.007693	0.007167	0.03942	< 0.01
unclassified_Bacteria	0.038268	0.007889	0.044782	0.011069	0.178928	0.393641	-
Methylomirabilota	0.029507	0.006847	0.02856	0.007287	0.786337	0.823782	-
Myxococcota	0.025439	0.006039	0.020277	0.003244	0.048042	0.176153	-
Chloroflexi	0.021232	0.00543	0.015581	0.009446	0.145439	0.399958	-
Nitrospirota	0.017587	0.003089	0.016313	0.003861	0.461539	0.564103	-
Verrucomicrobiota	0.009575	0.003048	0.007517	0.00309	0.18761	0.375221	-
Fibrobacterota	0.006903	0.003465	0.004995	0.002606	0.223817	0.41033	-
Patescibacteria	0.003291	0.003252	0.002925	0.002314	0.795019	0.795019	-
Dadabacteria	0.001289	0.000887	0.000098	0.000278	0.002464	0.027101	< 0.01
Firmicutes	0.001082	0.001151	0.003079	0.003072	0.089128	0.280116	-
Desulfobacterota	0.001075	0.000775	0.000155	0.000289	0.006504	0.047693	< 0.01
Latescibacterota	0.000831	0.000568	0.001432	0.001424	0.260079	0.440134	-
Campylobacterota	0.000562	0.000245	0	0	0.00001	0.00023	< 0.001
Sumerlaeota	0.000291	0.000448	0.000098	0.000208	0.283784	0.445946	-
Bdellovibrionota	0.000272	0.000284	0.000412	0.000525	0.499666	0.578561	-
Entotheonellaeota	0.000175	0.000345	0.000048	0.000089	0.328279	0.451383	-
Deferribacterota	0.000044	0.000083	0	0	0.153211	0.374517	-

Table S2. ANOVA analysis of variance for bulk soils on genus level of bacteria.

genus	LBS (Mean)	LBS(Sd)	HBS (Mean)	HBS(Sd)	MultiGroup (p)	MultiGroup (p-corrected)	LBS::HBS (p)
Sphingomonas	0.069716	0.015265	0.05507	0.00813	0.028673	0.141088	-
unclassified_Vicinamibacteraceae	0.054486	0.009046	0.08713	0.022819	0.001237	0.021309	< 0.01
unclassified_Vicinamibacterales	0.052942	0.009033	0.077918	0.019041	0.003068	0.036576	< 0.01
unclassified_Gemmatimonadaceae	0.051161	0.007769	0.041729	0.006453	0.016412	0.097838	-
unclassified_Bacteria	0.038268	0.007889	0.044782	0.011069	0.178928	0.423416	-
RB41	0.030653	0.007815	0.012496	0.003371	0.000021	0.002209	< 0.001
uncultured_Firmicutes_bacterium	0.020176	0.003924	0.013461	0.003894	0.003004	0.03725	< 0.01
unclassified_Geminicoccaceae	0.019845	0.008709	0.018903	0.005811	0.799552	0.914616	-
uncultured_gamma_proteobacterium	0.018287	0.00536	0.034882	0.010353	0.000735	0.015186	< 0.001
MND1	0.018143	0.002564	0.024688	0.00678	0.016594	0.097062	-
Nitrospira	0.017587	0.003089	0.016313	0.003861	0.461539	0.671723	-
Lysobacter	0.017544	0.004989	0.024357	0.005379	0.016143	0.098127	-
P3OB_42	0.016624	0.003519	0.008644	0.002647	0.000102	0.00635	< 0.001
unclassified_Sphingomonadaceae	0.01604	0.005987	0.011552	0.002716	0.070834	0.246725	-
unclassified_Micrococcaceae	0.015864	0.012099	0.008605	0.015196	0.290439	0.555779	-
Altererythrobacter	0.015275	0.004302	0.012243	0.003359	0.129602	0.361952	-
unclassified_Azospirillales	0.013456	0.001991	0.010139	0.002792	0.012254	0.084417	-
Bryobacter	0.012913	0.003119	0.00962	0.001372	0.014847	0.093927	-
unclassified_Microscillaceae	0.011887	0.002555	0.013354	0.002651	0.26376	0.520799	-
Arenimonas	0.011567	0.002186	0.008728	0.001992	0.01385	0.093339	-
Massilia	0.01151	0.006751	0.010903	0.004774	0.835464	0.911951	-
Subgroup_10	0.010671	0.005278	0.007141	0.00074	0.081284	0.268064	-
unclassified_Blastocatellaceae	0.010441	0.002895	0.009448	0.002295	0.449645	0.660615	-
unclassified_Comamonadaceae	0.010206	0.002771	0.006994	0.002746	0.030081	0.143463	-
Iamia	0.009645	0.003051	0.006626	0.003078	0.0607	0.229477	-
unclassified_Chloroflexi	0.00917	0.003748	0.007692	0.005127	0.504372	0.704304	-
unclassified_Xanthobacteraceae	0.008878	0.00275	0.013974	0.003811	0.006092	0.051041	-
Dongia	0.008084	0.003139	0.007409	0.002431	0.631136	0.795335	-
uncultured_Acidobacteria_bacterium	0.008024	0.002033	0.008516	0.003035	0.697238	0.837767	-
Terrimonas	0.008004	0.002626	0.006117	0.000711	0.068404	0.243739	-
unclassified_Alphaproteobacteria	0.007966	0.001727	0.007959	0.002618	0.994473	0.994473	-
unclassified_Frankiales	0.007875	0.004974	0.002138	0.001714	0.007413	0.058922	-
unclassified_Acidimicrobiia	0.0074	0.001578	0.004403	0.001454	0.001039	0.020129	< 0.01
Devosia	0.007199	0.00183	0.00578	0.002099	0.157103	0.392757	-
unclassified_Rokubacteriales	0.00715	0.00317	0.010637	0.003221	0.040082	0.18008	-
Steroidobacter	0.00706	0.002931	0.008476	0.004475	0.447078	0.659972	-
possible_genus_04	0.006627	0.003425	0.004608	0.002157	0.172803	0.421803	-
Limibaculum	0.006571	0.003285	0.003863	0.001574	0.051101	0.208436	-
Ellin6067	0.006395	0.001664	0.007912	0.002146	0.121907	0.356521	-

Variovorax	0.006014	0.002349	0.004903	0.003359	0.43745	0.648849	-
unclassified_SC_I_84	0.006004	0.002439	0.009784	0.004337	0.039886	0.181832	-
Allorhizobium_Neorhizobium_Pararhizobium_Rhizobium	0.005694	0.002359	0.004092	0.001315	0.110411	0.342274	-
Pseudomonas	0.005552	0.006381	0.003802	0.002569	0.480726	0.686752	-
uncultured_soil_bacterium	0.005495	0.002078	0.005269	0.002024	0.823296	0.921378	-
Ilumatobacter	0.005458	0.00265	0.006343	0.001037	0.390775	0.605701	-
unclassified_IMCC26256	0.005393	0.002426	0.003447	0.002794	0.144852	0.383797	-
Acidibacter	0.005348	0.002787	0.008689	0.003547	0.046226	0.193651	-
unclassified_Gemmatimonadota	0.005071	0.002539	0.004066	0.001614	0.352649	0.594137	-
Pedobacter	0.00494	0.002494	0.004082	0.003319	0.552442	0.741372	-
unclassified_Actinomarinales	0.004827	0.00167	0.001855	0.001617	0.002065	0.033685	< 0.01
Nitrosospira	0.004808	0.002724	0.001274	0.000699	0.002876	0.037149	< 0.01
Halomonas	0.004687	0.008898	0.000429	0.000758	0.198782	0.437038	-
unclassified_Rhodanobacteraceae	0.004629	0.00103	0.002636	0.000888	0.000708	0.015676	< 0.001
unclassified_Longimicrobiaceae	0.00454	0.001857	0.002981	0.003241	0.235811	0.487343	-
Nocardioides	0.004287	0.004986	0.003298	0.002728	0.626026	0.795361	-
Adhaeribacter	0.004241	0.001964	0.002396	0.001311	0.040162	0.177859	-
Bradyrhizobium	0.004165	0.001276	0.005612	0.001568	0.053391	0.212194	-
Reyranella	0.00402	0.000905	0.004236	0.001508	0.721711	0.857205	-
uncultured_Acidobacteriales_bacterium	0.003964	0.001193	0.00463	0.002737	0.516778	0.715184	-
Phenylobacterium	0.003837	0.001448	0.003909	0.001109	0.910338	0.956627	-
unclassified_Chitinophagaceae	0.003729	0.002083	0.00744	0.003374	0.014428	0.093178	-
unclassified_Subgroup_17	0.003701	0.00109	0.009981	0.003583	0.000152	0.006722	< 0.001
unclassified_Saprospiraceae	0.003494	0.002095	0.002785	0.001617	0.450981	0.659453	-
Luteimonas	0.003454	0.002144	0.002387	0.001338	0.244648	0.498953	-
unclassified_Elsterales	0.003395	0.000865	0.002065	0.001107	0.013991	0.09228	-
Pseudoxanthomonas	0.003386	0.002566	0.005502	0.002993	0.137265	0.373266	-
unclassified_Steroidobacteraceae	0.003312	0.001854	0.006851	0.002631	0.00554	0.05204	-
BIyi10	0.003113	0.001544	0.00323	0.00182	0.887849	0.952364	-
unclassified_Xanthomonadaceae	0.003004	0.002039	0.003152	0.001067	0.856168	0.93127	-
unclassified_Microbacteriaceae	0.002995	0.001643	0.003209	0.002136	0.819127	0.923379	-
unclassified_Subgroup_7	0.002948	0.002684	0.003905	0.002026	0.424647	0.64215	-
Nordella	0.002941	0.00108	0.001643	0.000938	0.018924	0.106662	-
unclassified_Microtrichales	0.002851	0.00049	0.001897	0.001075	0.029664	0.143684	-
Luteolibacter	0.002778	0.00226	0.001853	0.001687	0.35928	0.602037	-
Flavisolibacter	0.002774	0.001804	0.002958	0.001254	0.812824	0.922987	-
unclassified_TRA3_20	0.002746	0.001269	0.002335	0.001445	0.541109	0.735718	-
unclassified_Devosiaceae	0.002626	0.001426	0.001402	0.001414	0.096425	0.305018	-
unclassified_Anaerolineaceae	0.002624	0.001798	0.00146	0.00114	0.137541	0.370764	-
Gemmatimonas	0.002608	0.001423	0.002837	0.001604	0.759886	0.882265	-
Phyllobacterium	0.002599	0.001693	0.003897	0.003522	0.339062	0.58394	-

Chryseolinea	0.002468	0.001644	0.005992	0.003085	0.009153	0.069203	-
Chthoniobacter	0.002451	0.000678	0.001108	0.0007	0.00112	0.020422	< 0.01
unclassified_Rhizobiaceae	0.002427	0.001016	0.001897	0.001923	0.480357	0.689402	-
uncultured_Actinomycetales_bacterium	0.002425	0.001664	0.000604	0.001454	0.030552	0.143503	-
uncultured_Acidobacterium_sp.	0.002391	0.000934	0.006405	0.00214	0.000125	0.006484	< 0.001
Dyadobacter	0.002361	0.001436	0.002484	0.002517	0.902023	0.951112	-
Ohtaekwangia	0.002313	0.00124	0.001707	0.001288	0.338841	0.586819	-
uncultured_Sorangiineae_bacterium	0.002305	0.00165	0.001289	0.000686	0.12694	0.361023	-
unclassified_A4b	0.002239	0.001461	0.001952	0.001266	0.673555	0.835208	-
Vicinamibacter	0.002238	0.001294	0.001554	0.000497	0.180948	0.421758	-
Paracoccus	0.002212	0.001562	0.000821	0.001915	0.119682	0.353346	-
JGI_0001001_H03	0.002209	0.001282	0.000568	0.000687	0.005684	0.050345	-
Flavitalea	0.002148	0.001453	0.001481	0.000352	0.226568	0.474568	-
Mesorhizobium	0.002125	0.001408	0.002162	0.0013	0.956291	0.965635	-
Aeromicrobium	0.002014	0.001541	0.00358	0.002612	0.147303	0.386983	-
Ramlibacter	0.001961	0.001074	0.001824	0.000478	0.74375	0.870047	-
unclassified_Ilumatobacteraceae	0.001938	0.001044	0.001789	0.000406	0.711202	0.851246	-
SWB02	0.001912	0.00081	0.002254	0.001289	0.516938	0.712226	-
Pontibacter	0.001885	0.001209	0.001447	0.000687	0.3816	0.603551	-
uncultured_Chloroflexi_bacterium	0.001854	0.001063	0.000657	0.000682	0.015792	0.097913	-
unclassified_BIrii41	0.001782	0.000896	0.001601	0.001404	0.752745	0.877259	-
Flavobacterium	0.001755	0.001566	0.00151	0.001242	0.72836	0.858523	-
Flaviaesturariibacter	0.001742	0.00127	0.000068	0.000126	0.002156	0.031827	< 0.01
uncultured_bacterium_gp6	0.00174	0.001477	0.002533	0.000804	0.197895	0.438195	-
Phaselicystis	0.001737	0.000984	0.002179	0.00123	0.42372	0.643888	-
Pedomicrobium	0.001717	0.000667	0.001232	0.000652	0.151548	0.385081	-
TM7a	0.001687	0.002083	0.000946	0.001457	0.414642	0.633198	-
Candidatus_Solibacter	0.001684	0.000837	0.001596	0.000731	0.82122	0.922384	-
Lechevalieria	0.001612	0.002002	0.00174	0.001729	0.889746	0.951108	-
uncultured_Holophaga_sp.	0.001603	0.000348	0.001518	0.000854	0.786163	0.902632	-
Cellvibrio	0.001595	0.001368	0.001292	0.001587	0.678426	0.834571	-
Puia	0.001558	0.001241	0.000103	0.000291	0.005635	0.051376	-
Haliangium	0.00154	0.000849	0.00438	0.002426	0.004833	0.048327	< 0.01
Brevundimonas	0.001495	0.001032	0.001772	0.00085	0.558642	0.740082	-
Lacibacter	0.001479	0.001059	0.000727	0.000841	0.129413	0.364708	-
Ensifer	0.001475	0.001207	0.001144	0.001538	0.62655	0.792778	-
YC_ZSS_LKJ147	0.001457	0.001314	0.00027	0.000408	0.027415	0.137074	-
bacterium_WX65	0.001417	0.000628	0.003759	0.001335	0.000273	0.007706	< 0.001
uncultured_Nitrosomonadales_bacterium	0.001417	0.00038	0.000449	0.000422	0.000165	0.00567	< 0.001
unclassified_Rhizobiales	0.001402	0.00065	0.001788	0.000669	0.246763	0.49673	-
uncultured_Acidobacteriaceae_bacterium	0.001397	0.001278	0.000348	0.000532	0.04765	0.196954	-

Blastococcus	0.001371	0.001696	0.000701	0.000657	0.311843	0.558795	-
CL500_29_marine_group	0.001331	0.000958	0.000526	0.000528	0.052556	0.21159	-
unclassified_Pedosphaeraceae	0.001314	0.00081	0.001953	0.001048	0.17768	0.426982	-
uncultured_Alphaproteobacteria_bacterium	0.001296	0.000568	0.000873	0.000649	0.171704	0.422446	-
Limnobacter	0.001255	0.000855	0.000859	0.000475	0.264892	0.519724	-
unclassified_Sutterellaceae	0.001239	0.001038	0.003081	0.002596	0.068044	0.245274	-
Woeseia	0.001215	0.001436	0.001366	0.000858	0.799705	0.911428	-
uncultured_Bacteroidetes_bacterium	0.001207	0.001049	0.000425	0.000592	0.083145	0.271314	-
Caenimonas	0.0012	0.001704	0.001345	0.000868	0.830725	0.913208	-
Stenotrophobacter	0.0012	0.000659	0.000477	0.000547	0.027407	0.139283	-
uncultured_actinobacterium	0.001196	0.001211	0.000087	0.000246	0.022878	0.122281	-
unclassified_B1_7BS	0.001147	0.00089	0.001519	0.000985	0.426474	0.641781	-
Marmoricola	0.001138	0.001645	0.001222	0.001734	0.919798	0.956837	-
unclassified_KF_JG30_B3	0.001103	0.000465	0.001825	0.000858	0.044494	0.188947	-
unclassified_Sphingobacteriaceae	0.00109	0.000965	0.000355	0.000375	0.062097	0.231927	-
Ellin6055	0.001068	0.001747	0.003229	0.002728	0.067711	0.246945	-
Sphingopyxis	0.001025	0.000952	0.001314	0.001304	0.605615	0.782252	-
Edaphobaculum	0.001013	0.000648	0.000656	0.000435	0.207535	0.446778	-
Promicromonospora	0.001012	0.001329	0.00245	0.00242	0.143415	0.383263	-
unclassified_Nocardioidaceae	0.000979	0.001312	0.000348	0.000689	0.242869	0.498606	-
Luteitalea	0.000938	0.000968	0.003189	0.002137	0.012024	0.084717	-
Aridibacter	0.000934	0.000703	0.000593	0.000668	0.323073	0.575589	-
Algoriphagus	0.00092	0.002129	0.000096	0.00027	0.295817	0.562597	-
unclassified_Acidobacteriales	0.000904	0.00045	0.000059	0.000168	0.00016	0.006182	< 0.001
unclassified_Desulfovibrionaceae	0.000897	0.000682	0	0	0.0021	0.032549	< 0.01
Tabrizicola	0.000895	0.001015	0.000085	0.00024	0.044253	0.190535	-
OLB13	0.000883	0.000686	0.000603	0.000738	0.430171	0.644217	-
Ahniella	0.000879	0.000678	0.002587	0.001245	0.002784	0.037528	< 0.01
Mucilaginibacter	0.000871	0.000518	0.000893	0.000619	0.935744	0.963723	-
Sphingoaurantiacus	0.000862	0.000501	0.000353	0.000325	0.027125	0.140146	-
unclassified_Intrasporangiaceae	0.000845	0.000944	0.00106	0.001081	0.668013	0.831663	-
unclassified_11_24	0.000844	0.000426	0.000987	0.000662	0.598199	0.775906	-
unclassified_Latescibacteraceae	0.000831	0.000568	0.001432	0.001424	0.260079	0.516825	-
uncultured_beta_proteobacterium	0.000826	0.001177	0.001155	0.000586	0.485883	0.690935	-
unclassified_Solirubrobacteraceae	0.000824	0.001055	0.001546	0.001405	0.246003	0.498438	-
Hirschia	0.000818	0.000315	0.000866	0.000335	0.761632	0.880993	-
Microvirga	0.000817	0.000449	0.000518	0.000258	0.119162	0.355194	-
mle1_7	0.000803	0.00097	0.000984	0.000743	0.675712	0.834545	-
Stenotrophomonas	0.000771	0.001162	0.00101	0.000944	0.650902	0.813628	-
unclassified_Defluviicoccales	0.000769	0.000623	0.000339	0.000375	0.110536	0.339269	-
unclassified_Reyranellaceae	0.00076	0.000645	0.000827	0.000606	0.827023	0.92222	-

Pseudorhodoferax	0.000752	0.000664	0.000576	0.00065	0.590175	0.771959	-
Ferruginibacter	0.000731	0.001136	0.001452	0.001019	0.190673	0.428324	-
uncultured_Desulfovirga_sp.	0.00072	0.000501	0.000085	0.00024	0.005237	0.050733	-
unclassified_DS_100	0.000719	0.000463	0.000519	0.000452	0.382207	0.601442	-
unclassified_WX65	0.000714	0.000294	0	0	0.000006	0.001709	< 0.001
Ellin517	0.000709	0.000686	0.000641	0.000573	0.829774	0.918679	-
unclassified_Oxalobacteraceae	0.000705	0.000805	0.000679	0.000659	0.942916	0.964699	-
Bacillus	0.000701	0.001081	0.002782	0.003028	0.072172	0.243187	-
Roseimicrobium	0.0007	0.000597	0.000424	0.000535	0.334503	0.582561	-
Bosea	0.000692	0.0006	0.001129	0.000585	0.150153	0.384689	-
uncultured_Nitrospirae_bacterium	0.000649	0.000566	0.000006	0.000017	0.005952	0.051257	-
Methylotenera	0.000641	0.000471	0.001025	0.000898	0.278674	0.53993	-
unclassified_Dadabacteriales	0.00064	0.000696	0.000092	0.00026	0.05371	0.210759	-
Blastocatella	0.000632	0.000431	0.00009	0.000168	0.004533	0.048452	< 0.01
unclassified_Pseudomonadales	0.000595	0.000749	0.000522	0.000615	0.830615	0.916337	-
uncultured_Chlorobiales_bacterium	0.000575	0.000751	0.000129	0.000364	0.147745	0.384881	-
Noviherbaspirillum	0.000572	0.000342	0.000155	0.000438	0.043816	0.19131	-
Helicobacter	0.000562	0.000245	0	0	0.00001	0.00162	< 0.001
Amaricoccus	0.000556	0.000867	0.001016	0.000724	0.257135	0.514269	-
unclassified_Dehalococcoidia	0.000547	0.000845	0.000868	0.001471	0.582493	0.765139	-
unclassified_Caulobacteraceae	0.00053	0.000584	0.000378	0.000435	0.556502	0.740411	-
Polycyclovorans	0.000522	0.001018	0.000976	0.001053	0.38114	0.605915	-
Caulobacter	0.000512	0.000971	0.001122	0.000803	0.181471	0.419821	-
unclassified_Actinobacteriota	0.000496	0.000466	0.000982	0.000716	0.114159	0.343585	-
uncultured_bacterium_259	0.000467	0.000477	0.000949	0.00075	0.130416	0.360972	-
Lautropia	0.000463	0.0008	0.000768	0.000425	0.351313	0.59512	-
unclassified_LWQ8	0.000463	0.001006	0.000217	0.000305	0.518775	0.711594	-
MM2	0.000455	0.000385	0.000203	0.000379	0.195708	0.436471	-
Kribbella	0.000431	0.000664	0.00052	0.000622	0.781334	0.900422	-
unclassified_Gammaproteobacteria	0.00043	0.000536	0.000453	0.000609	0.935836	0.960626	-
Polaromonas	0.000406	0.000485	0.000635	0.000581	0.389847	0.607299	-
uncultured_Candidatus_Saccharibacteria_bacterium	0.000402	0.001206	0	0	0.362439	0.582156	-
Agromyces	0.000401	0.00056	0.002404	0.000859	0.000037	0.002899	< 0.001
Hydrogenophaga	0.0004	0.000509	0.000443	0.000539	0.866646	0.939371	-
Arthrobacter	0.000397	0.000629	0.000924	0.0009	0.178388	0.425386	-
uncultured_Burkholderiaceae_bacterium	0.00039	0.000373	0.000624	0.000171	0.124889	0.361827	-
Saccharothrix	0.000388	0.000483	0.000431	0.000559	0.868482	0.938082	-
unclassified_Saccharimonadales	0.000382	0.000584	0.000293	0.000224	0.690159	0.83574	-
Taibaiella	0.000343	0.000334	0.00022	0.000485	0.548013	0.738627	-
uncultured_Sphingobium_sp.	0.000342	0.000689	0.000059	0.000168	0.278084	0.542177	-
unclassified_Sandaracinaceae	0.000339	0.000476	0.000532	0.000433	0.398412	0.614467	-

Gaiella	0.000334	0.00045	0.00141	0.000918	0.006872	0.056064	-
Tagaea	0.000328	0.000547	0	0	0.111612	0.339212	-
unclassified_Kapabacteriales	0.000327	0.000322	0.000128	0.00026	0.185009	0.421713	-
Candidatus_Alysiosphaera	0.00031	0.000417	0.000218	0.000247	0.596081	0.776408	-
uncultured_Iamiaceae_bacterium	0.000306	0.000329	0.000396	0.000284	0.554685	0.741174	-
unclassified_env.OPS_17	0.000297	0.000626	0.000181	0.000513	0.684986	0.836006	-
unclassified_Myxococcaceae	0.000294	0.000454	0	0	0.087588	0.279922	-
Sumerlaea	0.000291	0.000448	0.000098	0.000208	0.283784	0.546416	-
Chryseobacterium	0.000286	0.000417	0.000168	0.000346	0.538156	0.734927	-
unclassified_KI89A_clade	0.000282	0.000311	0	0	0.021831	0.118727	-
Kaistia	0.000278	0.000382	0.000496	0.000458	0.301228	0.565943	-
unclassified_AKYG1722	0.000278	0.000426	0	0	0.085531	0.276193	-
unclassified_Fibrobacteraceae	0.000276	0.000393	0.000387	0.000503	0.616048	0.789153	-
OM27_clade	0.000272	0.000284	0.000412	0.000525	0.499666	0.700889	-
unclassified_SBR1031	0.000271	0.000305	0.000052	0.000097	0.071473	0.243478	-
Inquilinus	0.000269	0.000297	0.000339	0.000256	0.616359	0.786302	-
Sandaracinus	0.000264	0.000245	0.000223	0.000209	0.714424	0.851813	-
Sorangium	0.000264	0.000359	0.000448	0.000426	0.348149	0.596278	-
Rhodococcus	0.000262	0.000471	0.000397	0.000866	0.690377	0.83275	-
unclassified_NS11_12_marine_group	0.000259	0.000314	0.000104	0.00012	0.209534	0.447969	-
unclassified_0319_7L14	0.000258	0.000566	0.000198	0.00056	0.829069	0.921188	-
unclassified_Thalassobaculales	0.000258	0.000154	0.00019	0.000117	0.328994	0.579478	-
unclassified_Rhodobacteraceae	0.000255	0.000389	0.000786	0.000732	0.076778	0.255927	-
UTBCD1	0.000252	0.000511	0.000293	0.00083	0.90158	0.95389	-
Candidatus_Koribacter	0.00024	0.000312	0.000021	0.00006	0.070233	0.247413	-
unclassified_Solimonadaceae	0.000239	0.000354	0.000209	0.000536	0.894711	0.953129	-
Solirubrobacter	0.000238	0.000315	0.000345	0.000576	0.636355	0.798664	-
unclassified_Methyloligellaceae	0.000232	0.000286	0.00093	0.000719	0.016763	0.09623	-
unclassified_Gaiellales	0.000208	0.000372	0.000419	0.000675	0.431274	0.642763	-
Falsirhodobacter	0.000206	0.000364	0.000062	0.000121	0.304036	0.547972	-
Umezawaea	0.000206	0.000342	0.000314	0.000889	0.738092	0.866699	-
Ruminococcus	0.0002	0.000294	0.000269	0.000249	0.610457	0.785235	-
Bauldia	0.000197	0.000402	0.000098	0.000222	0.543655	0.735952	-
unclassified_Acetobacteraceae	0.000186	0.000187	0.000184	0.000262	0.984086	0.987271	-
Opitutus	0.00018	0.000199	0.00042	0.000112	0.008702	0.06744	-
Longimicrobium	0.000176	0.00025	0.000184	0.000261	0.948454	0.967174	-
Mycobacterium	0.000176	0.000288	0.000576	0.000868	0.209972	0.44583	-
unclassified_Entotheonellaceae	0.000175	0.000345	0.000048	0.000089	0.328279	0.581522	-
unclassified_Desulfobacterota	0.000169	0.000265	0.000155	0.000289	0.918249	0.958441	-
Rhizorhapis	0.000161	0.000484	0	0	0.362439	0.594477	-
Aquabacterium	0.000155	0.000241	0.000135	0.000258	0.871304	0.937862	-
Verticiella	0.000155	0.000167	0.000122	0.000169	0.685683	0.833576	-

Verrucomicrobium	0.000152	0.000247	0.000168	0.000246	0.896673	0.951948	-
Methylobacillus	0.00015	0.000199	0.000373	0.000488	0.225521	0.475589	-
Roseomonas	0.000147	0.000268	0.000196	0.000301	0.728174	0.86158	-
Acidovorax	0.000136	0.000278	0.000045	0.000127	0.412816	0.63353	-
unclassified_Burkholderiales	0.000134	0.000403	0.000026	0.000048	0.462765	0.670361	-
Lactobacillus	0.00013	0.00022	0.000006	0.000017	0.134069	0.367799	-
Pajaroellobacter	0.000128	0.000254	0.000516	0.000516	0.063276	0.233517	-
unclassified_Micromonosporaceae	0.000127	0.000256	0.000805	0.000554	0.00482	0.049803	< 0.01
Thermomonas	0.000119	0.000356	0.000112	0.000317	0.96814	0.974427	-
Cellulomonas	0.000115	0.000346	0.000133	0.000376	0.920685	0.954557	-
Escherichia_Shigella	0.000115	0.000187	0	0	0.101919	0.319141	-
Akkermansia	0.000114	0.000165	0	0	0.071106	0.244922	-
Odoribacter	0.00011	0.00013	0	0	0.030732	0.142195	-
unclassified_Ardenticatenales	0.000109	0.00017	0.000018	0.000052	0.168172	0.417068	-
unclassified_Fodinicurvataceae	0.000108	0.000188	0.000036	0.000103	0.350141	0.596394	-
Dokdonella	0.000107	0.000211	0.000101	0.000186	0.951633	0.967234	-
Lacunisphaera	0.000104	0.000233	0.000044	0.000066	0.489877	0.690281	-
Paenarthrobacter	0.000098	0.000294	0.000112	0.000214	0.915733	0.959044	-
Methylobacterium_Methylorubrum	0.000094	0.00016	0.000042	0.00012	0.467584	0.674191	-
Rhizobacter	0.000088	0.000185	0.000397	0.000625	0.175422	0.42485	-
uncultured_prokaryote	0.000086	0.00021	0.000155	0.000438	0.680189	0.833434	-
Piscinibacter	0.000079	0.000168	0.000175	0.000271	0.388939	0.608945	-
Aurantisolimonas	0.000077	0.00023	0.000319	0.000536	0.235697	0.490377	-
Nannocystis	0.000075	0.000224	0.000309	0.000659	0.32941	0.576933	-
Glycomyces	0.000071	0.000143	0.000263	0.000513	0.297408	0.562174	-
Skermanella	0.000068	0.000153	0.00014	0.000324	0.559277	0.737769	-
uncultured_Caldilineaceae_bacterium	0.000068	0.000203	0.000049	0.000139	0.831848	0.911211	-
Pelagibius	0.000067	0.000137	0	0	0.186603	0.422241	-
Microbacterium	0.000063	0.000188	0	0	0.362439	0.600835	-
unclassified_PAUC26f	0.000063	0.000188	0.000651	0.000361	0.000652	0.016837	< 0.001
unclassified_Micropepsaceae	0.000059	0.000178	0.000079	0.000151	0.814307	0.921297	-
Dactylosporangium	0.000054	0.000161	0.000397	0.000238	0.003106	0.035663	< 0.01
uncultured_Candidatus_Rokubactera_ bacterium	0.000049	0.000146	0.000702	0.000364	0.000166	0.005152	< 0.001
unclassified_Promicromonosporaceae	0.000047	0.000098	0.000138	0.000148	0.150032	0.387584	-
unclassified_Mitochondria	0.000045	0.000118	0.000168	0.00019	0.125062	0.358973	-
Mucispirillum	0.000044	0.000083	0	0	0.153211	0.386143	-
Fluviicola	0.000043	0.00013	0	0	0.362439	0.604065	-
Hyphomicrobium	0.000042	0.000127	0.000514	0.000305	0.000686	0.016359	< 0.001
unclassified_Acidobacteriota	0.000038	0.000113	0.000446	0.000322	0.002768	0.039006	< 0.01
unclassified_Rhizobiales_Incertae_Se dis	0.000038	0.000113	0.000115	0.000326	0.512343	0.712225	-
uncultured_Acidimicrobiia_bacterium	0.000038	0.000114	0.000168	0.000251	0.180204	0.423206	-

unclassified_Subgroup_2	0.000035	0.00008	0.000032	0.000091	0.954593	0.967071	-
uncultured_Nitrosomonadaceae_bacterium	0.000035	0.000105	0	0	0.362439	0.579155	-
unclassified_67_14	0.000029	0.00006	0.000032	0.000091	0.935137	0.966308	-
Bacteroides	0.000026	0.000053	0	0	0.184434	0.423515	-
Ligilactobacillus	0.000026	0.000079	0.000006	0.000017	0.489567	0.692995	-
Nonomuraea	0.000026	0.000079	0.000521	0.000572	0.02103	0.116415	-
Romboutsia	0.000022	0.000065	0	0	0.362439	0.591348	-
unclassified_YM_S32_TM7_50_20	0.000016	0.000049	0.00141	0.002023	0.055673	0.215731	-
Desulfovibrio	0.000009	0.000019	0	0	0.205623	0.448896	-
Parabacteroides	0.000006	0.000018	0	0	0.362439	0.597639	-
unclassified_Muribaculaceae	0.000003	0.000009	0	0	0.362439	0.588252	-
unclassified_Peptococcaceae	0.000003	0.000008	0	0	0.362439	0.585188	-
Clostridium_sensu_stricto_1	0	0	0.000006	0.000018	0.303673	0.5671	-
Faecalibacterium	0	0	0.000003	0.000009	0.303673	0.563704	-
JTB255_marine_benthic_group	0	0	0.000067	0.00019	0.303673	0.560348	-
Oscillibacter	0	0	0.000006	0.000017	0.303673	0.557033	-
Salinispora	0	0	0.000495	0.000505	0.00984	0.070941	-
Streptomyces	0	0	0.001283	0.001296	0.009323	0.06881	-
Streptosporangium	0	0	0.000344	0.000411	0.023609	0.124047	-
Thauera	0	0	0.000324	0.000739	0.20671	0.448113	-
unclassified_Amb_16S_1323	0	0	0.000134	0.000198	0.058304	0.223138	-
unclassified_Roseiflexaceae	0	0	0.000033	0.000092	0.303673	0.553756	-
unclassified_S085	0	0	0.000061	0.000174	0.303673	0.550518	-
unclassified_SAR202_clade	0	0	0.000532	0.000474	0.00411	0.045499	< 0.01

Table S3. ANOVA analysis of variance for rhizosphere soils on phylum level of bacteria.

phylum	LRS(Mean)	LRS(Sd)	HRS(Mean)	HRS(Sd)	multiGroup(p)	multiGroup(p-corrected)	LRS::HRS(p)
Acidobacteriota	0.14084	0.021296	0.173259	0.048796	0.089758	0.282097	-
Actinobacteriota	0.132776	0.019627	0.143126	0.050674	0.578116	0.79491	-
Bacteroidota	0.093315	0.012755	0.083535	0.015505	0.17405	0.425454	-
Bdellovibrionota	0.000744	0.00057	0.000632	0.000589	0.69586	0.850495	-
Campylobacterota	0	0	0.000003	0.000008	0.303673	0.556733	-
Chloroflexi	0.018997	0.006493	0.012706	0.006228	0.060254	0.265116	-
Dadabacteria	0.000594	0.000779	0.000059	0.000166	0.076813	0.281648	-
Desulfobacterota	0.000003	0.00001	0	0	0.362439	0.613358	-
Entotheonellaeota	0.000172	0.000285	0	0	0.109266	0.300482	-
Fibrobacterota	0.011942	0.005627	0.011596	0.007922	0.917824	0.917824	-
Firmicutes	0.001034	0.00126	0.007525	0.003258	0.000056	0.001231	< 0.001
Gemmatimonadota	0.044772	0.009673	0.032153	0.006572	0.007277	0.080047	-
Latescibacterota	0.000284	0.00032	0.000538	0.000575	0.270605	0.54121	-
Methylomirabilota	0.012445	0.00428	0.011991	0.003889	0.822828	0.905111	-
Myxococcota	0.030363	0.005682	0.023371	0.006417	0.030763	0.225594	-
Nitrospirota	0.008578	0.001781	0.008851	0.00241	0.792391	0.917505	-
Patescibacteria	0.010954	0.008081	0.009438	0.003565	0.632226	0.818175	-
Planctomycetota	0.000128	0.000285	0	0	0.225889	0.496955	-
Proteobacteria	0.455067	0.023302	0.447352	0.024088	0.512641	0.805579	-
Sumerlaeota	0.000096	0.000196	0.000082	0.000159	0.876243	0.917969	-
Verrucomicrobiota	0.013114	0.005462	0.007509	0.004304	0.034309	0.1887	-
unclassified_Bacteria	0.023783	0.007186	0.026276	0.008532	0.522875	0.766883	-

Table S4. ANOVA analysis of variance for rhizosphere soils on genus level of bacteria.

genus	LRS (Mean)	LRS(Sd)	HRS (Mean)	HRS(Sd)	multiGroup (p)	multiGroup(p-corrected)	LRS::HRS (p)
Sphingomonas	0.06718	0.014865	0.052617	0.00984	0.03306	0.209157	-
unclassified_Vicinamibacteraceae	0.038014	0.007884	0.061142	0.018645	0.003915	0.093361	-
unclassified_Vicinamibacterales	0.036572	0.007028	0.053926	0.017307	0.014275	0.138285	-
unclassified_Gemmatimonadaceae	0.034913	0.007187	0.025142	0.00514	0.006159	0.095472	-
unclassified_Bacteria	0.023783	0.007186	0.026276	0.008532	0.522875	0.726866	-
Altererythrobacter	0.021229	0.006308	0.019482	0.004167	0.516657	0.724723	-
Massilia	0.020806	0.015247	0.029917	0.013341	0.212199	0.494599	-
Lysobacter	0.018665	0.00482	0.021405	0.008592	0.422839	0.636311	-
Devosia	0.017352	0.003747	0.015167	0.005982	0.375014	0.608662	-
RB41	0.01719	0.0074	0.005626	0.003351	0.001037	0.045914	< 0.01
unclassified_Sphingomonadaceae	0.016126	0.005428	0.01325	0.002687	0.195512	0.469835	-
unclassified_Microbacteriaceae	0.015426	0.010933	0.012984	0.007069	0.598102	0.788986	-
unclassified_Microscillaceae	0.015007	0.004025	0.014097	0.002647	0.595373	0.792127	-
unclassified_Micrococcaceae	0.014976	0.008305	0.016184	0.011712	0.80779	0.900773	-
MND1	0.013986	0.00375	0.01309	0.005237	0.688063	0.849799	-
Nocardioides	0.013656	0.006074	0.016873	0.00751	0.344377	0.613546	-
unclassified_Geminicoccaceae	0.013159	0.005955	0.014455	0.007005	0.685746	0.85374	-
uncultured_gamma_proteobacterium	0.012153	0.003996	0.020524	0.006236	0.004508	0.093166	-
Variovorax	0.011617	0.003733	0.009028	0.001977	0.100311	0.345516	-
Pedobacter	0.011343	0.006738	0.009682	0.003519	0.541957	0.746697	-
Arenimonas	0.011254	0.003125	0.008755	0.003032	0.115944	0.366762	-
unclassified_Comamonadaceae	0.011076	0.003308	0.007768	0.003615	0.067508	0.279033	-
unclassified_Chloroflexi	0.01005	0.004815	0.00675	0.003689	0.137379	0.394329	-
P3OB_42	0.009997	0.003469	0.005012	0.002689	0.005079	0.087478	-
Allorhizobium_Neorhizobium_Pararhizobium_Rhizobium	0.0099	0.003912	0.011618	0.001467	0.260998	0.550404	-
Iamia	0.009126	0.002745	0.006328	0.003124	0.067996	0.277353	-
Dyadobacter	0.008963	0.005073	0.009519	0.006795	0.849893	0.91165	-
Bryobacter	0.008823	0.002367	0.008451	0.002743	0.768333	0.878905	-
Nitrospira	0.008578	0.001781	0.008851	0.00241	0.792391	0.890004	-
unclassified_Azospirillales	0.008556	0.001522	0.006543	0.002281	0.046952	0.255351	-
uncultured_Firmicutes_bacterium	0.008432	0.002979	0.005335	0.002173	0.028683	0.197597	-
possible_genus_04	0.008429	0.004398	0.009428	0.005919	0.69626	0.843128	-
Terrimonas	0.008159	0.002798	0.00758	0.001978	0.63322	0.814516	-
Acidibacter	0.007574	0.004783	0.008743	0.002978	0.560866	0.76933	-
unclassified_Xanthobacteraceae	0.00756	0.002366	0.008835	0.002583	0.304916	0.562642	-
Phyllobacterium	0.007545	0.004699	0.010288	0.004683	0.2475	0.536539	-
Aeromicrobium	0.007384	0.001812	0.009257	0.0026	0.102264	0.340882	-
Dongia	0.007369	0.002924	0.005452	0.00194	0.137233	0.397592	-
unclassified_Rhizobiaceae	0.007291	0.002444	0.006956	0.003974	0.834976	0.908219	-

unclassified_Blastocatellaceae	0.007062	0.002556	0.005277	0.002419	0.161394	0.435063	-
Pseudomonas	0.007044	0.003322	0.01272	0.009548	0.113989	0.364293	-
BIyi10	0.006186	0.002786	0.004782	0.002252	0.275238	0.561341	-
uncultured_Sorangiineae_bacterium	0.006127	0.003258	0.003395	0.001165	0.040568	0.232889	-
unclassified_Acidimicrobiia	0.00602	0.002559	0.002524	0.001568	0.004482	0.099246	-
Luteimonas	0.005858	0.003261	0.00363	0.002409	0.133995	0.395606	-
Promicromonospora	0.005846	0.0038	0.013688	0.01581	0.168446	0.435151	-
Steroidobacter	0.005753	0.002323	0.006015	0.002617	0.829836	0.909007	-
Luteolibacter	0.005751	0.004435	0.002426	0.002065	0.071808	0.289097	-
Pseudoxanthomonas	0.005659	0.003169	0.012234	0.005185	0.00598	0.097574	-
unclassified_BIrii41	0.005515	0.004465	0.004597	0.005153	0.699163	0.843348	-
Cellvibrio	0.005483	0.003655	0.005978	0.00427	0.800225	0.895558	-
Subgroup_10	0.005439	0.002611	0.005381	0.001334	0.955573	0.96491	-
TM7a	0.005362	0.005238	0.004559	0.002274	0.694597	0.851087	-
Flavobacterium	0.00495	0.004673	0.004442	0.002407	0.786354	0.886436	-
unclassified_Alphaproteobacteria	0.004862	0.000979	0.00433	0.000881	0.25926	0.550483	-
Phenylobacterium	0.004598	0.001742	0.004452	0.000534	0.823395	0.90515	-
unclassified_Rhodanobacteraceae	0.004593	0.00112	0.003142	0.001142	0.018519	0.140023	-
uncultured_Acidobacteria_bacterium	0.00453	0.00103	0.00522	0.002122	0.397909	0.626151	-
Ellin6067	0.004489	0.001509	0.004076	0.002018	0.637294	0.809677	-
unclassified_IMCC26256	0.004481	0.002467	0.001249	0.000855	0.00315	0.081384	-
Ohtaekwangia	0.004429	0.00155	0.003982	0.001742	0.583801	0.783456	-
Mesorhizobium	0.004424	0.001876	0.004175	0.001351	0.760922	0.883468	-
Mucilaginibacter	0.004385	0.003006	0.002836	0.001969	0.234882	0.520095	-
Limibaculum	0.00422	0.002544	0.003069	0.001782	0.302978	0.602073	-
unclassified_Devosiaceae	0.004166	0.000973	0.002665	0.001341	0.017686	0.144283	-
Adhaeribacter	0.004077	0.003851	0.001698	0.001299	0.117619	0.364619	-
unclassified_Saprospiraceae	0.004059	0.001848	0.001741	0.001661	0.016278	0.144179	-
Reyranella	0.003996	0.001713	0.003676	0.000801	0.636773	0.812344	-
Brevundimonas	0.003941	0.0019	0.003457	0.000911	0.522584	0.729734	-
Bradyrhizobium	0.003882	0.001557	0.004367	0.001026	0.46713	0.686305	-
Ilumatobacter	0.003881	0.001613	0.004201	0.001584	0.686662	0.85146	-
Stenotrophomonas	0.003809	0.002697	0.004321	0.004031	0.759768	0.885444	-
Halomonas	0.003791	0.005619	0.000097	0.000139	0.083774	0.316707	-
unclassified_Longimicrobiaceae	0.003722	0.001637	0.00219	0.001755	0.08218	0.314515	-
unclassified_Gemmatimonadota	0.003633	0.001552	0.002296	0.000864	0.047936	0.247672	-
unclassified_Frankiales	0.00363	0.003438	0.001846	0.001449	0.19377	0.472982	-
Lechevalieria	0.003581	0.003795	0.004882	0.001729	0.388279	0.623661	-
unclassified_Fibrobacteraceae	0.003513	0.003824	0.002168	0.00224	0.398413	0.623777	-
Flavisolibacter	0.003503	0.001358	0.003264	0.001025	0.691654	0.850845	-
Sphingopyxis	0.00329	0.001799	0.003754	0.002105	0.631166	0.815256	-
Nitrosospira	0.003289	0.002361	0.000473	0.000373	0.00462	0.089503	-

Chthoniobacter	0.003252	0.001252	0.00125	0.00103	0.002787	0.078531	-
Paracoccus	0.003132	0.001613	0.001614	0.002394	0.141904	0.399911	-
unclassified_Xanthomonadaceae	0.00313	0.001907	0.003195	0.001259	0.93594	0.954413	-
Caulobacter	0.003101	0.003039	0.003606	0.002799	0.727391	0.860653	-
unclassified_SC_I_84	0.003078	0.001804	0.005419	0.002233	0.030417	0.204981	-
uncultured_soil_bacterium	0.003054	0.000883	0.002755	0.001601	0.635316	0.813835	-
unclassified_Rokubacteriales	0.002965	0.001757	0.004317	0.001392	0.102058	0.343893	-
Lacibacter	0.00293	0.002184	0.001579	0.00106	0.133543	0.39806	-
unclassified_Actinomarinales	0.00286	0.001372	0.000746	0.000563	0.001043	0.040411	< 0.01
unclassified_Mitochondria	0.002806	0.0021	0.001543	0.000695	0.126272	0.387568	-
Phaselicystis	0.002771	0.001465	0.002869	0.000953	0.873679	0.921226	-
unclassified_TRA3_20	0.002771	0.000996	0.001075	0.000398	0.000426	0.026441	< 0.001
Nordella	0.002666	0.00122	0.001176	0.000903	0.012709	0.135858	-
uncultured_Acidobacteriales_bacterium	0.002646	0.001214	0.003228	0.001844	0.448869	0.665787	-
Chryseolinea	0.002601	0.002427	0.004757	0.001982	0.065273	0.281038	-
unclassified_Chitinophagaceae	0.002486	0.002271	0.007	0.003831	0.009	0.111601	-
unclassified_Subgroup_17	0.002444	0.00131	0.005224	0.0023	0.007159	0.105684	-
Rhodococcus	0.002385	0.001027	0.002301	0.000839	0.857467	0.916603	-
Algoriphagus	0.002375	0.005339	0.000365	0.000963	0.312171	0.569253	-
Pseudorhodoferax	0.00236	0.001403	0.002202	0.001418	0.820655	0.905349	-
unclassified_Ilumatobacteraceae	0.002307	0.000996	0.001755	0.001575	0.39554	0.628807	-
unclassified_Subgroup_7	0.002288	0.001916	0.003014	0.001686	0.422361	0.638692	-
uncultured_Actinomycetales_bacterium	0.002256	0.001341	0.00063	0.000895	0.011002	0.12632	-
Vicinamibacter	0.002247	0.001011	0.001672	0.00062	0.184317	0.457107	-
Marmoricola	0.002141	0.001224	0.000774	0.001128	0.030818	0.203264	-
Polaromonas	0.002103	0.002038	0.001497	0.001103	0.465794	0.687601	-
unclassified_Elsterales	0.002086	0.000695	0.001141	0.000587	0.008884	0.114752	-
uncultured_Acidobacterium_sp.	0.00205	0.000734	0.004454	0.002561	0.016301	0.140374	-
Blastococcus	0.002031	0.001355	0.001116	0.000483	0.091141	0.324755	-
unclassified_Saccharimonadales	0.002027	0.002264	0.001508	0.001377	0.583084	0.785896	-
unclassified_Steroidobacteraceae	0.002011	0.001473	0.004151	0.002206	0.031096	0.200827	-
Haliangium	0.001952	0.001167	0.002505	0.001389	0.386138	0.623451	-
Mycobacterium	0.001931	0.001648	0.003212	0.001718	0.137577	0.391274	-
unclassified_A4b	0.001915	0.001462	0.002607	0.001864	0.405171	0.628015	-
Streptomyces	0.001833	0.001	0.004817	0.001399	0.000129	0.019921	< 0.001
Polycyclovorans	0.001812	0.001269	0.001288	0.001194	0.395937	0.626227	-
Gemmatimonas	0.001805	0.00101	0.002103	0.001092	0.567796	0.775404	-
unclassified_Microtrichales	0.001789	0.000715	0.001415	0.000874	0.347576	0.615707	-
Bosea	0.001687	0.000819	0.001909	0.000882	0.597664	0.791777	-
CL500_29_marine_group	0.001651	0.000722	0.000625	0.000628	0.007234	0.101931	-
Flavitalea	0.001631	0.001167	0.000677	0.000749	0.067078	0.281004	-

Agromyces	0.001606	0.001304	0.004374	0.001422	0.000793	0.04095	< 0.001
Kribbella	0.001606	0.001385	0.001421	0.001055	0.763742	0.880149	-
unclassified_LWQ8	0.001598	0.002089	0.002465	0.001974	0.394398	0.630223	-
unclassified_Sphingobacteriaceae	0.001565	0.001406	0.001369	0.001256	0.76727	0.88094	-
Ramlibacter	0.001561	0.001078	0.001148	0.000622	0.356956	0.618192	-
unclassified_Solirubrobacteraceae	0.001535	0.000987	0.002209	0.001102	0.202997	0.476736	-
unclassified_Micromonosporaceae	0.001496	0.000733	0.001575	0.001611	0.895834	0.935046	-
Caenimonas	0.001485	0.001083	0.001457	0.000972	0.956943	0.963157	-
Ellin6055	0.001471	0.00188	0.001651	0.00251	0.868431	0.921964	-
unclassified_Rhizobiales	0.00145	0.000531	0.001106	0.000455	0.174709	0.440323	-
unclassified_Intrasporangiaceae	0.001437	0.001596	0.003734	0.001943	0.017274	0.144731	-
Puia	0.001427	0.001429	0.000422	0.000598	0.08492	0.313394	-
uncultured_bacterium_gp6	0.001397	0.000856	0.001553	0.000519	0.661243	0.826554	-
unclassified_Pedosphaeraceae	0.001384	0.000656	0.001401	0.001074	0.969107	0.969107	-
Chryseobacterium	0.001377	0.001077	0.002661	0.003724	0.336609	0.606679	-
OLB13	0.001338	0.000585	0.000683	0.000984	0.111295	0.359391	-
Candidatus_Solibacter	0.001329	0.00088	0.000784	0.000531	0.149465	0.417426	-
Woeseia	0.001323	0.001614	0.00109	0.001421	0.757141	0.885713	-
Tabrizicola	0.00132	0.001434	0.000203	0.000376	0.050023	0.246144	-
Saccharothrix	0.001297	0.001237	0.001167	0.001003	0.81646	0.903938	-
unclassified_Dehalococcoidia	0.001289	0.001624	0.001213	0.001156	0.913834	0.944295	-
Solirubrobacter	0.001269	0.000835	0.001481	0.000945	0.629293	0.816238	-
unclassified_Sandaracinaceae	0.001265	0.001312	0.000891	0.000623	0.474853	0.684671	-
unclassified_Gaiellales	0.001251	0.001263	0.001053	0.001038	0.731974	0.862783	-
Cellulomonas	0.00124	0.001423	0.000852	0.000873	0.515321	0.726133	-
Gaiella	0.001237	0.000899	0.001873	0.001265	0.246791	0.54259	-
Methylobacillus	0.001226	0.000782	0.000804	0.000816	0.292698	0.589197	-
uncultured_Chloroflexi_bacterium	0.001221	0.000989	0.000228	0.000395	0.018111	0.140363	-
Edaphobaculum	0.001211	0.000646	0.00107	0.00062	0.653281	0.819907	-
Microbacterium	0.0012	0.000993	0.000342	0.000679	0.058074	0.268699	-
uncultured_Acidobacteriaceae_bacterium	0.001197	0.001049	0.000443	0.000702	0.106285	0.350514	-
uncultured_Holophaga_sp.	0.001168	0.000622	0.001079	0.000679	0.78146	0.884133	-
uncultured_Sphingobium_sp.	0.001126	0.001873	0.000108	0.000305	0.150665	0.417018	-
uncultured_actinobacterium	0.001117	0.001168	0.000131	0.000369	0.037578	0.228417	-
Flaviaesturariibacter	0.001099	0.001445	0.000044	0.000125	0.058201	0.265328	-
Kaistia	0.001097	0.001346	0.000766	0.00038	0.513794	0.727288	-
Methylotenera	0.001086	0.001049	0.002037	0.00194	0.220709	0.499414	-
unclassified_Nocardioidaceae	0.001085	0.000969	0.001161	0.000947	0.873129	0.923788	-
Sorangium	0.001031	0.001435	0.001939	0.001516	0.223994	0.503176	-
Umezawaea	0.001029	0.00104	0.000861	0.001324	0.774022	0.882157	-
Ensifer	0.000998	0.00079	0.001511	0.00201	0.489184	0.695629	-

Hydrogenophaga	0.000971	0.001049	0.00134	0.001018	0.473961	0.686578	-
Taibaiella	0.000968	0.000622	0.000839	0.001655	0.830465	0.906493	-
SWB02	0.000958	0.000574	0.001302	0.000851	0.339375	0.608129	-
unclassified_Actinobacteriota	0.000954	0.00052	0.000994	0.001319	0.933402	0.958128	-
Limnobacter	0.000935	0.000844	0.000466	0.000344	0.16398	0.430794	-
unclassified_Anaerolineaceae	0.000924	0.000543	0.000293	0.000375	0.014923	0.140185	-
JGI_0001001_H03	0.000919	0.000634	0.000314	0.000506	0.047691	0.254899	-
Lautropia	0.000914	0.000897	0.000958	0.000882	0.920655	0.948183	-
Bacillus	0.000894	0.001226	0.007219	0.003186	0.000057	0.017822	< 0.001
Nonomuraea	0.000883	0.000943	0.001002	0.000738	0.777351	0.882706	-
Luteitalea	0.000863	0.000849	0.002433	0.00162	0.02234	0.161055	-
Pontibacter	0.000833	0.000619	0.000855	0.000522	0.937759	0.953132	-
bacterium_WX65	0.000814	0.000315	0.002097	0.000754	0.000295	0.022859	< 0.001
unclassified_Sutterellaceae	0.000782	0.000562	0.001938	0.001398	0.037036	0.229623	-
Methylobacterium_Methylorubrum	0.000781	0.000537	0.000449	0.000339	0.15494	0.421328	-
Ahniella	0.000763	0.00039	0.001685	0.001298	0.05979	0.268621	-
unclassified_DS_100	0.000753	0.000834	0.000627	0.000404	0.704264	0.846209	-
Roseimicrobium	0.000751	0.000531	0.000705	0.000385	0.841203	0.908616	-
Fluviicola	0.000747	0.000732	0.000388	0.000518	0.267355	0.560001	-
OM27_clade	0.000744	0.00057	0.000632	0.000589	0.69586	0.849278	-
unclassified_0319_7L14	0.000736	0.000574	0.00016	0.000453	0.03796	0.2263	-
unclassified_Reyranellaceae	0.000735	0.000864	0.000655	0.000669	0.836032	0.906189	-
uncultured_Nitrosomonadales_bacterium	0.00071	0.000505	0.000176	0.000204	0.013806	0.138058	-
Ferruginibacter	0.000708	0.001142	0.001427	0.001017	0.192533	0.473691	-
MM2	0.000706	0.000702	0.000354	0.000573	0.278374	0.564026	-
Aquabacterium	0.000702	0.000863	0.002162	0.002296	0.095799	0.333681	-
uncultured_Candidatus_Saccharibacteria_bacterium	0.0007	0.002099	0	0	0.362439	0.594477	-
uncultured_Alphaproteobacteria_bacterium	0.000697	0.0008	0.000284	0.000455	0.218755	0.502325	-
Aridibacter	0.000692	0.000689	0.000391	0.0006	0.353972	0.616468	-
Noviherbaspirillum	0.000689	0.000595	0.000562	0.00077	0.706379	0.845473	-
unclassified_Acidobacteriales	0.000685	0.000498	0	0	0.001495	0.051497	-
Pedomicrobium	0.000663	0.000548	0.00053	0.000396	0.580413	0.785712	-
Microvirga	0.000615	0.000603	0.000164	0.000304	0.076017	0.298295	-
uncultured_beta_proteobacterium	0.000595	0.000801	0.000464	0.000665	0.719061	0.854057	-
unclassified_Oxalobacteraceae	0.000588	0.001114	0.000548	0.000837	0.935362	0.956971	-
Dactylosporangium	0.000581	0.000555	0.000803	0.000581	0.431765	0.646605	-
unclassified_KF_JG30_B3	0.00058	0.000675	0.000871	0.000551	0.349125	0.614935	-
unclassified_NS11_12_marine_group	0.00058	0.000586	0.000099	0.00014	0.039111	0.228763	-
unclassified_Pseudomonadales	0.000561	0.000599	0.000215	0.000307	0.163181	0.43236	-
unclassified_Methyloligellaceae	0.000558	0.000434	0.000948	0.000625	0.152124	0.417332	-
Dokdonella	0.000554	0.000795	0.000314	0.000317	0.438012	0.652806	-

Sphingoaurantiacus	0.000548	0.0005	0.00038	0.000289	0.417421	0.634316	-
unclassified_env.OPS_17	0.000545	0.001202	0	0	0.220417	0.502422	-
Skermanella	0.000541	0.000427	0.000287	0.000373	0.212636	0.491919	-
YC_ZSS_LKJ147	0.000505	0.000863	0.00032	0.000583	0.617668	0.80792	-
unclassified_Burkholderiales	0.000499	0.001003	0.000144	0.000282	0.349734	0.612529	-
Amaricoccus	0.000498	0.000842	0.0012	0.000466	0.054371	0.259306	-
mle1_7	0.000482	0.000572	0.000613	0.000572	0.645626	0.816914	-
Roseomonas	0.000476	0.000403	0.000441	0.000322	0.84956	0.914457	-
Stenotrophobacter	0.000465	0.000609	0.00029	0.000321	0.478067	0.686115	-
unclassified_Defluviicoccales	0.000465	0.000399	0	0	0.004969	0.090619	-
uncultured_prokaryote	0.000454	0.00053	0.000238	0.000674	0.471109	0.685652	-
Pajaroellobacter	0.000434	0.000832	0.000861	0.000681	0.268871	0.559396	-
uncultured_Chlorobiales_bacterium	0.000434	0.00066	0.000289	0.000409	0.601095	0.789574	-
Hyphomicrobium	0.00043	0.000341	0.000635	0.000362	0.247118	0.539484	-
Lacunisphaera	0.000416	0.000511	0.000052	0.000096	0.066579	0.282733	-
Inquilinus	0.0004	0.000402	0.000303	0.000266	0.570519	0.775706	-
Acidovorax	0.000396	0.000497	0.000354	0.000796	0.897532	0.933674	-
Ellin517	0.000378	0.000456	0.000668	0.000248	0.130784	0.393621	-
unclassified_B1_7BS	0.000378	0.000576	0.000412	0.000398	0.888623	0.930653	-
unclassified_Micropepsaceae	0.000373	0.000309	0.000104	0.000296	0.088463	0.318878	-
Glycomyces	0.000372	0.000597	0.001114	0.001429	0.173711	0.441396	-
Piscinibacter	0.000371	0.000407	0.000209	0.000307	0.37193	0.606832	-
Verticiella	0.000367	0.000263	0.000272	0.000192	0.412257	0.629555	-
Bauldia	0.000364	0.000491	0.000098	0.000277	0.196209	0.467882	-
unclassified_Caulobacteraceae	0.000364	0.000615	0.000337	0.00023	0.907578	0.940967	-
unclassified_Dadabacteriales	0.000364	0.000527	0.000053	0.00015	0.128231	0.389723	-
uncultured_bacterium_259	0.000363	0.000472	0.000963	0.00079	0.072724	0.289032	-
Sandaracinus	0.000353	0.000376	0.000712	0.000431	0.086981	0.317225	-
uncultured_Bacteroidetes_bacterium	0.000341	0.000699	0.000041	0.000116	0.251277	0.540945	-
Rhizobacter	0.000331	0.000618	0.001559	0.001135	0.013025	0.134594	-
Hirschia	0.00033	0.00037	0.000609	0.000258	0.09534	0.335856	-
unclassified_Gammaproteobacteria	0.00032	0.000417	0.000308	0.000318	0.949171	0.961579	-
Falsirhodobacter	0.000318	0.000533	0.000509	0.000673	0.52451	0.725884	-
uncultured_Desulfovirga_sp.	0.000304	0.000398	0	0	0.048458	0.246263	-
unclassified_Promicromonosporaceae	0.000299	0.00036	0.000253	0.000236	0.763691	0.883374	-
Sphingobium	0.000298	0.000491	0.000198	0.000386	0.64887	0.817682	-
Verrucomicrobium	0.000298	0.000895	0.000216	0.00034	0.810377	0.900419	-
Candidatus_Koribacter	0.000289	0.000364	0	0	0.041192	0.232172	-
unclassified_KI89A_clade	0.000286	0.000348	0.000041	0.000116	0.078255	0.303239	-
unclassified_11_24	0.000285	0.000231	0.000554	0.0003	0.055142	0.259002	-
UTBCD1	0.000284	0.000598	0.000196	0.000554	0.757037	0.888945	-
unclassified_Latescibacteraceae	0.000284	0.00032	0.000538	0.000575	0.270605	0.555546	-

unclassified_Solimonadaceae	0.000268	0.000409	0.000095	0.000268	0.325034	0.589242	-
unclassified_Myxococcaceae	0.000265	0.000449	0	0	0.116861	0.365928	-
Arthrobacter	0.000253	0.000401	0.005572	0.007519	0.050373	0.243992	-
Rhizorhapis	0.000252	0.000599	0	0	0.25365	0.542286	-
Salinispora	0.000239	0.000493	0.000852	0.000664	0.045781	0.253432	-
unclassified_WX65	0.000234	0.000227	0	0	0.010826	0.129079	-
uncultured_Nitrospirae_bacterium	0.00023	0.000362	0.000006	0.000017	0.101636	0.346233	-
unclassified_AKYG1722	0.000225	0.000207	0	0	0.007933	0.106923	-
uncultured_Burkholderiaceae_bacterium	0.000213	0.000328	0.000205	0.000283	0.957882	0.960982	-
Nannocystis	0.000199	0.000427	0.000351	0.000815	0.628989	0.819271	-
Longimicrobium	0.000193	0.000318	0.000102	0.000176	0.48445	0.692072	-
Tagaea	0.000193	0.000386	0	0	0.179762	0.449405	-
unclassified_Thalassobaculales	0.000191	0.000232	0.000137	0.000153	0.583817	0.780101	-
Thermomonas	0.000185	0.000555	0	0	0.362439	0.60733	-
unclassified_Rhodobacteraceae	0.000184	0.000295	0.000664	0.000593	0.047695	0.250599	-
unclassified_Acetobacteraceae	0.000183	0.000385	0.000297	0.000209	0.470469	0.68795	-
Candidatus_Alysiosphaera	0.000181	0.000285	0.000226	0.000194	0.71365	0.850891	-
unclassified_Kapabacteriales	0.00018	0.000221	0.000054	0.000152	0.194308	0.470589	-
unclassified_Entotheonellaceae	0.000172	0.000285	0	0	0.109266	0.356553	-
uncultured_Acidimicrobiia_bacterium	0.000164	0.000265	0.000068	0.000191	0.408391	0.626738	-
Pelagibius	0.000157	0.000322	0.000032	0.000091	0.306365	0.561971	-
Paenarthrobacter	0.000156	0.000343	0.00038	0.000462	0.26916	0.556264	-
Blastocatella	0.000145	0.000221	0	0	0.084619	0.316046	-
unclassified_Rhizobiales_Incertae_Sedis	0.000142	0.000223	0.000095	0.000268	0.696245	0.846415	-
unclassified_YM_S32_TM7_50_20	0.000142	0.000374	0.000798	0.000626	0.017858	0.141948	-
Duganella	0.000132	0.000267	0.001046	0.001024	0.020527	0.151508	-
Singulisphaera	0.000128	0.000285	0	0	0.225889	0.503781	-
Opitutus	0.000124	0.000191	0.000139	0.000157	0.865052	0.921533	-
Aurantisolimonas	0.000118	0.000202	0.000554	0.000958	0.200821	0.475226	-
Ruminococcus	0.000114	0.000172	0.000204	0.000258	0.406344	0.626699	-
unclassified_SBR1031	0.000109	0.000175	0.000017	0.000047	0.169131	0.43331	-
Sumerlaea	0.000096	0.000196	0.000082	0.000159	0.876243	0.920798	-
uncultured_Iamiaceae_bacterium	0.00009	0.000136	0.000168	0.000234	0.403765	0.62898	-
Streptosporangium	0.000045	0.000136	0.000603	0.000567	0.011706	0.129598	-
unclassified_PAUC26f	0.00004	0.00008	0.000462	0.000254	0.00026	0.026865	< 0.001
Klebsiella	0.000037	0.00011	0.00041	0.000768	0.168401	0.438692	-
unclassified_Acidobacteriota	0.000034	0.000101	0.000278	0.000162	0.001873	0.058057	-
Lactobacillus	0.000014	0.000043	0	0	0.362439	0.620752	-
unclassified_Acidobacteriaceae__Subgroup_1	0.000012	0.000037	0	0	0.362439	0.604065	-
Erysipelatoclostridium	0.000003	0.000009	0	0	0.362439	0.624201	-
Ligilactobacillus	0.000003	0.000009	0	0	0.362439	0.617341	-

Parabacteroides	0.000003	0.000009	0	0	0.362439	0.613968	-
Subdoligranulum	0.000003	0.000009	0	0	0.362439	0.610631	-
unclassified_Desulfovibrionaceae	0.000003	0.00001	0	0	0.362439	0.600835	-
unclassified_Oscillospiraceae	0.000003	0.000009	0	0	0.362439	0.597639	-
Bacteroides	0	0	0.000006	0.000017	0.303673	0.599609	-
Clostridium_sensu_stricto_1	0	0	0.000011	0.000017	0.063556	0.277498	-
Curtobacterium	0	0	0.000462	0.000687	0.060758	0.269071	-
Helicobacter	0	0	0.000003	0.000008	0.303673	0.595814	-
JTB255_marine_benthic_group	0	0	0.000045	0.000127	0.303673	0.592066	-
Lactococcus	0	0	0.000087	0.000166	0.13548	0.396214	-
Oscillibacter	0	0	0.000003	0.000008	0.303673	0.588366	-
Pedococcus_Phycicoccus	0	0	0.000091	0.000258	0.303673	0.584711	-
Serratia	0	0	0.001421	0.003928	0.293121	0.586243	-
Thauera	0	0	0.000215	0.000441	0.1622	0.433467	-
Virgisporangium	0	0	0.000029	0.000083	0.303673	0.581102	-
unclassified_67_14	0	0	0.000074	0.000104	0.048521	0.242607	-
unclassified_Amb_16S_1323	0	0	0.000217	0.000259	0.023663	0.166717	-
unclassified_Ardenticatenales	0	0	0.000008	0.000024	0.303673	0.577537	-
unclassified_Enterobacteriaceae	0	0	0.000031	0.000088	0.303673	0.574015	-
unclassified_Fodinicurvataceae	0	0	0.00002	0.000055	0.303673	0.570537	-
unclassified_SAR202_clade	0	0	0.000034	0.000095	0.303673	0.5671	-
unclassified_Subgroup_2	0	0	0.00001	0.000028	0.303673	0.563704	-
uncultured_Candidatus_Rokubactera_bacterium	0	0	0.000241	0.000267	0.015542	0.141709	-

Table S5. ANOVA analysis of variance for roots on phylum level of bacteria.

phylum	LR(Mean)	LR(Sd)	HR(Mean)	HR(Sd)	multiGroup(p)	multiGroup(p-corrected)	LR::HR(p)
Acidobacteriota	0.002232	0.003457	0.006151	0.006588	0.138971	0.509559	-
Actinobacteriota	0.013232	0.006645	0.014313	0.010308	0.798363	0.878199	-
Bacteroidota	0.068922	0.015785	0.057697	0.013356	0.136928	0.753105	-
Fibrobacterota	0.000432	0.001295	0.001654	0.002618	0.232956	0.512503	-
Firmicutes	0.002918	0.006434	0.006134	0.002936	0.214751	0.590566	-
Gemmatimonadota	0.00251	0.005694	0.006637	0.004907	0.132645	1.459098	-
Methylomirabilota	0.003737	0.004039	0.001828	0.003626	0.323949	0.593906	-
Myxococcota	0.013309	0.006578	0.011985	0.005816	0.668293	0.816803	-
Nitrospirota	0.019668	0.006592	0.023543	0.013269	0.449231	0.705935	-
Patescibacteria	0.017295	0.032539	0.009614	0.009477	0.530723	0.729745	-
Proteobacteria	0.855744	0.039134	0.860445	0.041682	0.813734	0.813734	-

Table S6. ANOVA analysis of variance for roots on genus level of bacteria.

genus	LR (Mean)	LR(Sd)	HR (Mean)	HR(Sd)	multiGroup (p)	multiGroup (p-corrected)	LR::HR (p)
Acidibacter	0.015696	0.00915	0.014728	0.009877	0.836585	0.976016	-
Acidovorax	0.002155	0.00458	0.010166	0.022841	0.317891	0.798804	-
Allorhizobium_Neorhizobium_Pararhizobium_Rhizobium	0.054474	0.039304	0.054064	0.01119	0.977704	1.008579	-
Altererythrobacter	0.032089	0.015295	0.026958	0.012707	0.466991	0.817235	-
Arenimonas	0.013753	0.009994	0.006749	0.003118	0.077419	0.632256	-
BIyi10	0.005082	0.004538	0.004732	0.00399	0.868647	0.978476	-
Bacillus	0.002918	0.006434	0.006134	0.002936	0.214751	0.725712	-
Bosea	0.002074	0.00258	0.00294	0.003078	0.537599	0.823198	-
Bradyrhizobium	0.01326	0.00477	0.014042	0.004479	0.733295	0.984424	-
Brevundimonas	0.008496	0.005346	0.007026	0.004998	0.568299	0.85682	-
Bryobacter	0.000529	0.001588	0.00439	0.004642	0.032698	0.801096	-
Caenimonas	0.006465	0.004402	0.006069	0.005904	0.876659	0.976279	-
Candidatus_Solibacter	0.000642	0.001926	0.000828	0.002343	0.859652	0.979604	-
Caulobacter	0.01731	0.030866	0.01575	0.016102	0.899873	0.990871	-
Cellvibrio	0.017291	0.026053	0.010253	0.008162	0.476333	0.818959	-
Chryseobacterium	0.002705	0.003409	0.0011	0.002176	0.272568	0.741989	-
Devosia	0.04063	0.009629	0.040605	0.00995	0.995771	1.006037	-
Dokdonella	0.001629	0.002165	0.000231	0.000655	0.100589	0.547651	-
Dongia	0.006329	0.004606	0.005819	0.004342	0.817798	0.977369	-
Duganella	0.004351	0.006898	0.011394	0.006882	0.052711	0.737956	-
Ensifer	0.00259	0.003568	0.000825	0.001233	0.204574	0.716009	-
Ferruginibacter	0.000727	0.001693	0.001628	0.002373	0.377273	0.786655	-
Flavobacterium	0.007239	0.012847	0.002812	0.002528	0.354754	0.790133	-
Hydrogenophaga	0.006003	0.006515	0.008892	0.004936	0.324108	0.774698	-
Hyphomicrobium	0.001252	0.003027	0.003777	0.004637	0.198495	0.720464	-
Ilumatobacter	0.000587	0.00176	0.00335	0.003991	0.078648	0.513832	-
Lacibacter	0.003539	0.003688	0.003054	0.002954	0.770619	0.968214	-
Lautropia	0.000069	0.000207	0.000596	0.001687	0.365172	0.777975	-
Lechevalieria	0.000476	0.001428	0.00023	0.00065	0.661302	0.912782	-
Luteimonas	0.016932	0.012591	0.010265	0.009148	0.236197	0.680803	-
Lysobacter	0.037639	0.026365	0.017091	0.008904	0.05362	0.656848	-
MND1	0.009857	0.007016	0.01555	0.010025	0.190926	0.719644	-
Massilia	0.040887	0.057864	0.048248	0.041246	0.769611	0.979505	-
Mesorhizobium	0.002767	0.002709	0.00432	0.00123	0.157827	0.644461	-
Methylobacillus	0.00504	0.003048	0.003288	0.002451	0.215018	0.702391	-
Methylophilus	0.000824	0.001267	0.001089	0.002085	0.75309	0.997336	-
Methylotenera	0.002155	0.003623	0.006687	0.006915	0.105506	0.544187	-
Mucilaginibacter	0.005409	0.004798	0.002018	0.002697	0.09828	0.566555	-
Nannocystis	0.001499	0.002389	0.000812	0.001613	0.503614	0.836512	-

Nitrospira	0.019668	0.006592	0.023543	0.013269	0.449231	0.830654	-
Nocardioides	0.00245	0.002768	0.004507	0.003769	0.215613	0.681616	-
Nordella	0.001738	0.002623	0.000642	0.001815	0.338318	0.789409	-
Ohtaekwangia	0	0	0.001575	0.002927	0.125827	0.616552	-
P3OB_42	0.001284	0.00236	0.002856	0.001821	0.148574	0.633056	-
Pajaroellobacter	0.002268	0.003824	0.003877	0.004808	0.454399	0.82465	-
Paracoccus	0.001179	0.002358	0.000285	0.000609	0.315295	0.813129	-
Pedobacter	0.011253	0.007486	0.013225	0.004794	0.533933	0.830562	-
Phenylobacterium	0.008126	0.003649	0.005416	0.003239	0.128238	0.598442	-
Phyllobacterium	0.035402	0.021717	0.040691	0.020542	0.614728	0.885932	-
Polaromonas	0.007742	0.007528	0.010227	0.005702	0.459558	0.818848	-
Pseudomonas	0.045982	0.026911	0.062148	0.036447	0.310875	0.823398	-
Pseudorhodoferax	0.012741	0.011251	0.009027	0.005447	0.410061	0.837208	-
Pseudoxanthomonas	0.039664	0.016014	0.065562	0.039305	0.08879	0.543836	-
Puia	0.000728	0.001533	0.003355	0.003793	0.07473	0.665775	-
Reyranella	0.0019	0.003847	0.001382	0.002675	0.755139	0.986714	-
Rhizobacter	0.005753	0.004728	0.00628	0.004247	0.813145	0.983805	-
Sorangium	0.000809	0.001341	0.001876	0.003803	0.44119	0.831474	-
Sphingobium	0.001755	0.00306	0.001173	0.00223	0.664101	0.903915	-
Sphingomonas	0.087066	0.035474	0.08688	0.022264	0.990043	1.010669	-
Sphingopyxis	0.009593	0.005682	0.019456	0.031177	0.364369	0.793514	-
Stenotrophomonas	0.012754	0.014162	0.014362	0.012499	0.808451	0.990353	-
Steroidobacter	0.001801	0.002607	0.002151	0.002014	0.763221	0.984154	-
Streptomyces	0.000804	0.001611	0.00046	0.0013	0.637342	0.892279	-
Tabrizicola	0.003474	0.00424	0.000497	0.001406	0.078339	0.548373	-
Terrimonas	0.019149	0.00654	0.017046	0.009116	0.589557	0.875403	-
Variovorax	0.059296	0.029414	0.035294	0.014583	0.054461	0.593017	-
Xanthomonas	0.00105	0.00209	0.001328	0.00301	0.825978	0.975251	-
possible_genus_04	0.000432	0.001295	0.001654	0.002618	0.232956	0.691809	-
unclassified_Actinomarinales	0.00338	0.002484	0.000677	0.00153	0.017953	0.879689	-
unclassified_Azospirillales	0.010816	0.004313	0.008568	0.006798	0.422501	0.828102	-
unclassified_BIrii41	0.001537	0.002032	0	0	0.050008	0.816801	-
unclassified_Burkholderiales	0.002972	0.008546	0	0	0.342638	0.780895	-
unclassified_Caulobacteraceae	0.005564	0.004037	0.003962	0.006067	0.526642	0.860182	-
unclassified_Chitinophagaceae	0.007873	0.004818	0.005999	0.004403	0.417667	0.835334	-
unclassified_Comamonadaceae	0.027103	0.008662	0.025748	0.013639	0.807833	1.002121	-
unclassified_Frankiales	0.00068	0.002041	0.000736	0.002082	0.956323	1.007739	-
unclassified_Geminicoccaceae	0.012479	0.004883	0.010717	0.008406	0.599493	0.876871	-
unclassified_Gemmatimonadaceae	0.00251	0.005694	0.006637	0.004907	0.132645	0.590875	-
unclassified_Methylophilaceae	0.001581	0.002695	0.000871	0.0017	0.532458	0.841627	-
unclassified_Microbacteriaceae	0.002262	0.003748	0.000504	0.001426	0.232157	0.710981	-
unclassified_Micromonosporaceae	0.002005	0.003433	0.002153	0.003134	0.927589	0.998942	-

unclassified_Microscillaceae	0.002979	0.004081	0.002656	0.002126	0.844	0.973082	-
unclassified_Oxalobacteraceae	0.002855	0.003176	0.002753	0.001946	0.938543	0.999752	-
unclassified_Rhizobiaceae	0.01989	0.006621	0.029588	0.006754	0.009225	0.904043	-
unclassified_Rhodobacteraceae	0.005207	0.006964	0.000479	0.001164	0.07821	0.589586	-
unclassified_SC_I_84	0.005649	0.003191	0.005651	0.002616	0.998743	0.998743	-
unclassified_Saccharimonadales	0.017295	0.032539	0.009614	0.009477	0.530723	0.852638	-
unclassified_Sphingobacteriaceae	0.005533	0.002786	0.002186	0.002597	0.022118	0.722523	-
unclassified_Sphingomonadaceae	0.028688	0.014635	0.020369	0.007123	0.165488	0.648712	-
unclassified_Steroidobacteraceae	0.001923	0.003286	0.001999	0.002196	0.956993	0.997716	-
unclassified_Xanthobacteraceae	0.011003	0.00489	0.017145	0.006492	0.042236	0.827819	-
unclassified_Xanthomonadaceae	0.004579	0.004245	0.003298	0.00304	0.490798	0.829279	-
uncultured_Acidobacteria_bacterium	0.001061	0.001844	0.000932	0.002636	0.907699	0.988383	-
uncultured_Chlorobiales_bacterium	0.001788	0.003604	0.001042	0.002138	0.617722	0.877344	-
uncultured_Firmicutes_bacterium	0.003737	0.004039	0.001828	0.003626	0.323949	0.793674	-
uncultured_Sorangiineae_bacterium	0.005912	0.004028	0.002563	0.002636	0.064141	0.628586	-
uncultured_gamma_proteobacterium	0.010638	0.003998	0.013111	0.004737	0.261219	0.731412	-
uncultured_soil_bacterium	0.001269	0.002139	0.002925	0.005672	0.427231	0.820954	-
